# Supplementary material for: Systematic assessment of prognostic molecular features across cancers
Source: Cell Genom. 2023 Feb 2;3(3):100262. doi: 10.1016/j.xgen.2023.100262 (PMC10025453; doi:10.1016/j.xgen.2023.100262)
Supplement: Document S1. Figures S1–S32 [file mmc1.pdf]

Cell Genomics, Volume *3*

Supplemental information

Systematic assessment of prognostic molecular features across cancers

Balaji Santhanam, Panos Oikonomou, and Saeed Tavazoie

Supplemental figures and legends

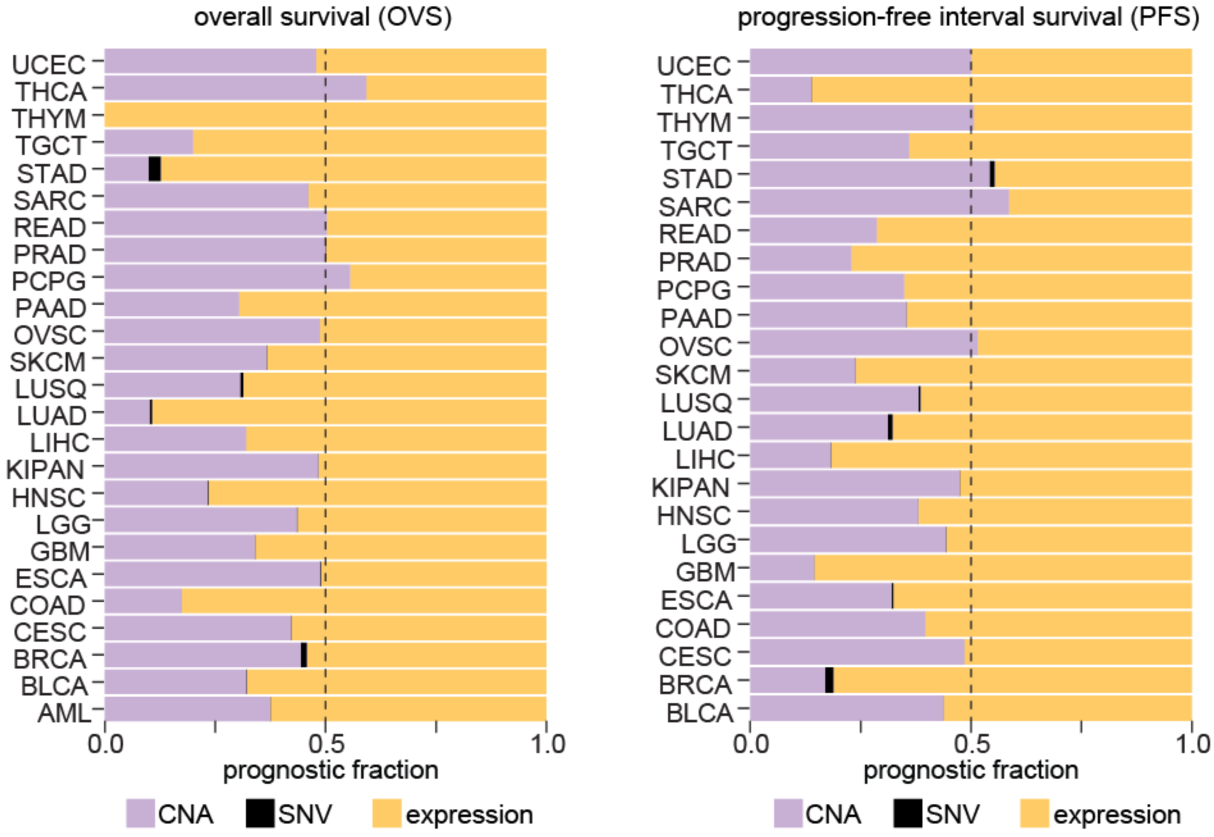

Figure S1. Prognosis specified by genomic and transcriptomic observations on individual genes, Related to Figure 1.

(a) Proportion of genes prognostic based on their copy-number aberrations (purple), mutation statuses (black) and expression changes (yellow) in each cohort (Y-axis) for overall survival (left panel) and progression-free interval survival (right panel).

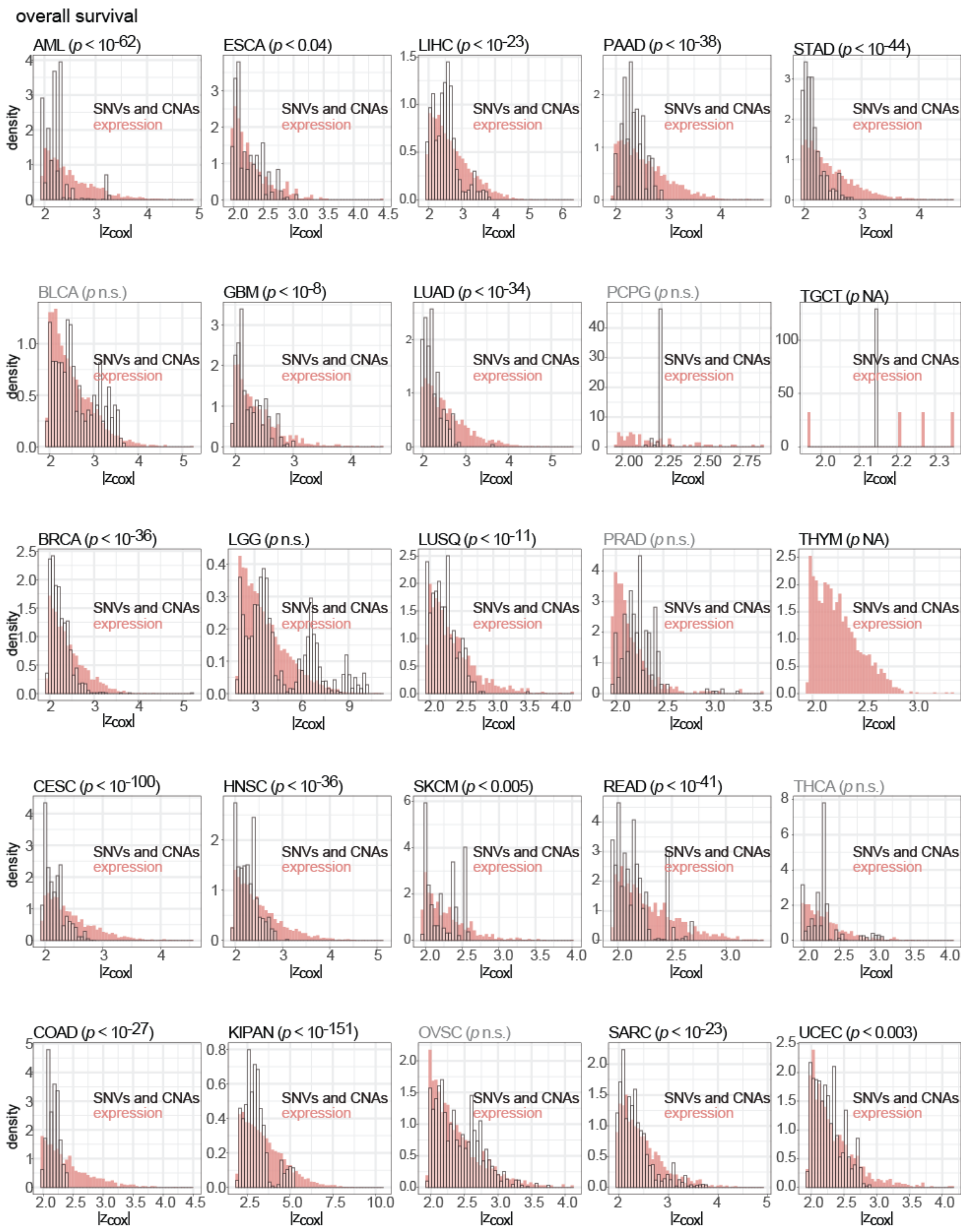
overall survival
AML (p < 10-62)
ESCA (p < 0.04)
LIHC (p < 10-23)
PAAD (p < 10-38)
STAD (p < 10-44)
BLCA (p n.s.)
GBM (p < 10-8)
LUAD (p < 10-34)
PCPG (p n.s.)
TGCT (p NA)
BRCA (p < 10-36)
LGG (p n.s.)
LUSQ (p < 10-11)
PRAD (p n.s.)
THYM (p NA)
CESC (p < 10-100)
HNSC (p < 10-36)
SKCM (p < 0.005)
READ (p < 10-41)
THCA (p n.s.)
COAD (p < 10-27)
KIPAN (p < 10-151)
OVSC (p n.s.)
SARC (p < 10-23)
UCEC (p < 0.003)
SNVs and CNAs
expression
density
|zcox|

Figure S2. Gene expression changes are more strongly prognostic for overall survival than either copy-number aberrations or mutations in individual genes, Related to Figure 1.

Distributions of absolute standardized significance scores (X-axis; Wald statistic) in patient stratifications based on gene expression changes or copy-number aberrations and mutation statuses are shown for each cohort in TCGA (color key indicated). For each gene's expression and copy-number, patients were stratified into groups with relatively high and low expression (positive and negative z-scored gene expression) and, high and low copy-number (positive and negative germline-subtracted segmented somatic copy-number) respectively, to quantify differences in overall survival. For mutations, patients were grouped into those with observed mutations and those with no observed mutations to quantify differences in overall survival. One-sided Mann-Whitney test *p*-values are indicated for each cohort.

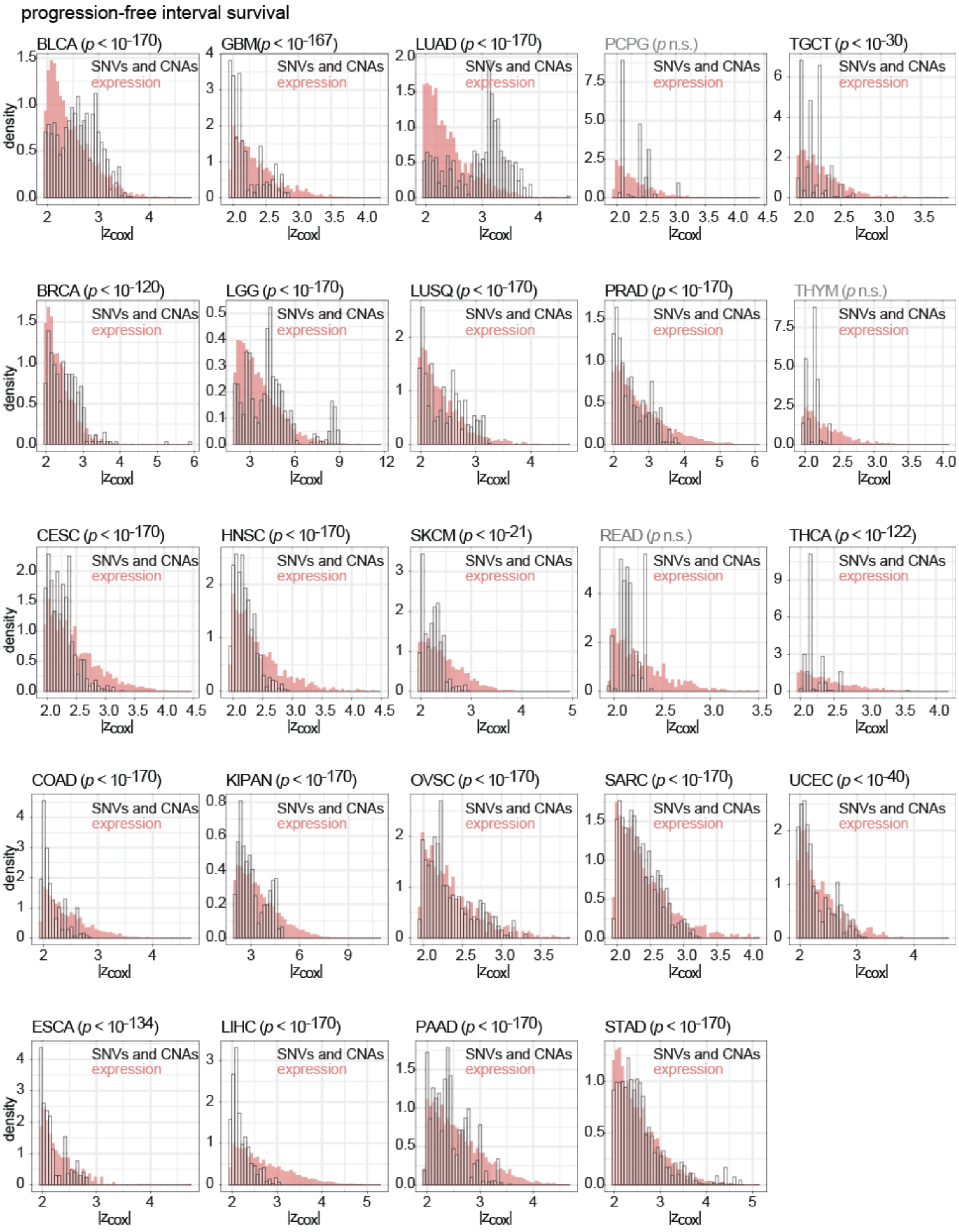
progression-free interval survival
BLCA (p < 10-170)
GBM(p < 10-167)
LUAD (p < 10-170)
PCPG (p n.s.)
TGCT (p < 10-30)
BRCA (p < 10-120)
LGG (p < 10-170)
LUSQ (p < 10-170)
PRAD (p < 10-170)
THYM (p n.s.)
CESC (p < 10-170)
HNSC (p < 10-170)
SKCM (p < 10-21)
READ (p n.s.)
THCA (p < 10-122)
COAD (p < 10-170)
KIPAN (p < 10-170)
OVSC (p < 10-170)
SARC (p < 10-170)
UCEC (p < 10-40)
ESCA (p < 10-134)
LIHC (p < 10-170)
PAAD (p < 10-170)
STAD (p < 10-170)
SNVs and CNAs
expression
density
|zcox|

Figure S3. Gene expression changes are more strongly prognostic for progression-free interval survival than either copy-number aberrations or mutations in individual genes, Related to Figure 1.

Distributions of absolute standardized significance scores (X-axis; Wald statistic) in patient stratifications based on gene expression changes or copy-number aberrations and mutation statuses are shown for each cohort in TCGA (color key indicated). For each gene's expression and copy-number, patients were stratified into groups with relatively high and low expression (positive and negative z-scored gene expression) and, high and low copy-number (positive and negative germline-subtracted segmented somatic copy-number) respectively, to quantify differences in progression-free interval survival. For mutations, patients were grouped into those with observed mutations and those with no observed mutations to quantify differences in progression-free interval survival. One-sided Mann-Whitney test *p*-values are indicated for each cohort.

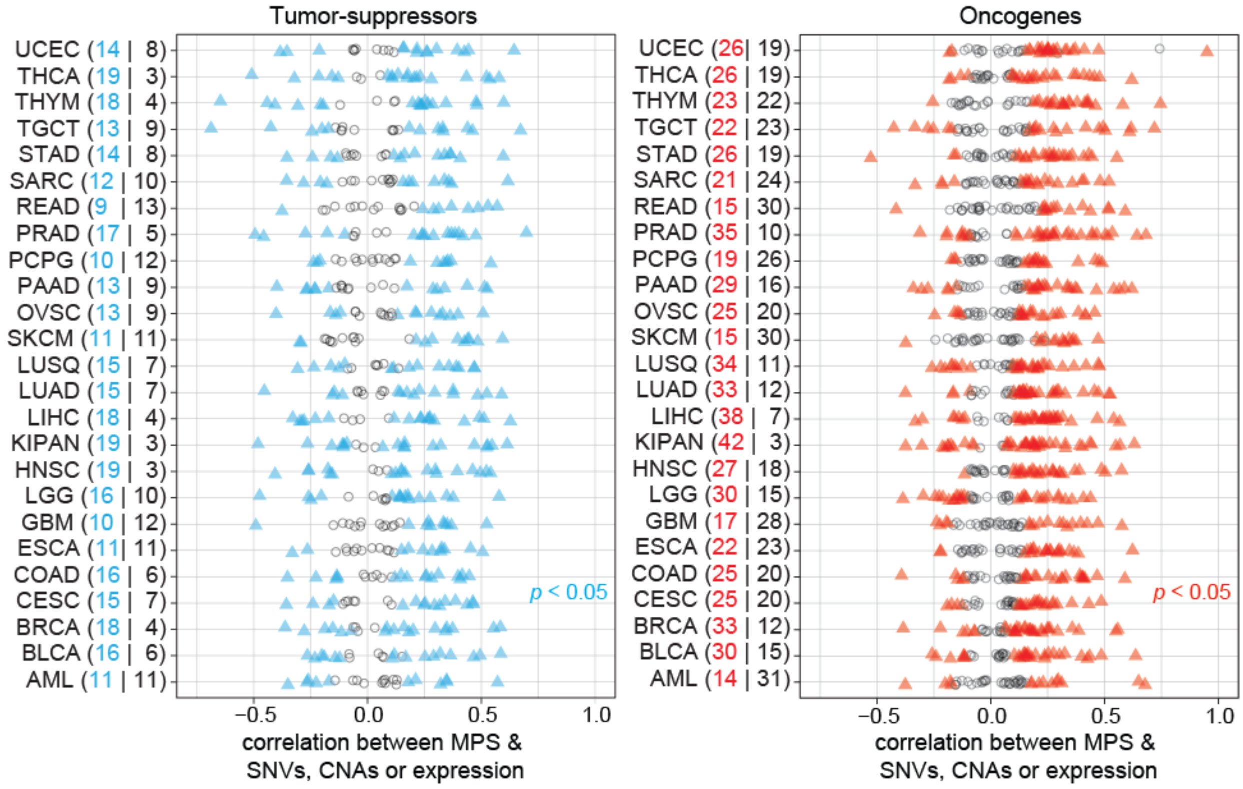

Tumor-suppressors
UCEC (14 | 8)
THCA (19 | 3)
THYM (18 | 4)
TGCT (13 | 9)
STAD (14 | 8)
SARC (12 | 10)
READ (9 | 13)
PRAD (17 | 5)
PCPG (10 | 12)
PAAD (13 | 9)
OVSC (13 | 9)
SKCM (11 | 11)
LUSQ (15 | 7)
LUAD (15 | 7)
LIHC (18 | 4)
KIPAN (19 | 3)
HNSC (19 | 3)
LGG (16 | 10)
GBM (10 | 12)
ESCA (11| 11)
COAD (16 | 6)
CESC (15 | 7)
BRCA (18 | 4)
BLCA (16 | 6)
AML (11 | 11)
p < 0.05
−0.5 0.0 0.5 1.0
correlation between MPS & SNVs, CNAs or expression
Oncogenes
UCEC (26| 19)
THCA (26 | 19)
THYM (23 | 22)
TGCT (22 | 23)
STAD (26 | 19)
SARC (21 | 24)
READ (15 | 30)
PRAD (35 | 10)
PCPG (19 | 26)
PAAD (29 | 16)
OVSC (25 | 20)
SKCM (15 | 30)
LUSQ (34 | 11)
LUAD (33 | 12)
LIHC (38 | 7)
KIPAN (42 | 3)
HNSC (27 | 18)
LGG (30 | 15)
GBM (17 | 28)
ESCA (22 | 23)
COAD (25 | 20)
CESC (25 | 20)
BRCA (33 | 12)
BLCA (30 | 15)
AML (14 | 31)
p < 0.05
−0.5 0.0 0.5 1.0
correlation between MPS & SNVs, CNAs or expression

Figure S4. Molecular measurements on genes encoding tumor-suppressors and oncogenes are largely correlated with perturbations in their associated modules, Related to Figure 1.

Distributions of correlations coefficients between module perturbations of 67 cancer-drivers[1] with mutations, copy-number aberrations or expression changes in genes encoding them across 25 cancers from TCGA (Y-axis). For every cohort, there are 22 points corresponding to tumor-suppressors (left panel) and 45 points corresponding to oncogenes[1]. Each dot represents the strongest correlation between module perturbations and each of the three single-gene measurements. Spearman's correlation coefficients were computed between copy-number aberrations and expression changes with module perturbations whereas Pearson's correlation coefficients were used for mutation statuses. Numbers of significant (p-value < 0.05; blue or red triangles) and not significant (p-value > 0.05; gray dots) correlation coefficients are indicated for each cohort.

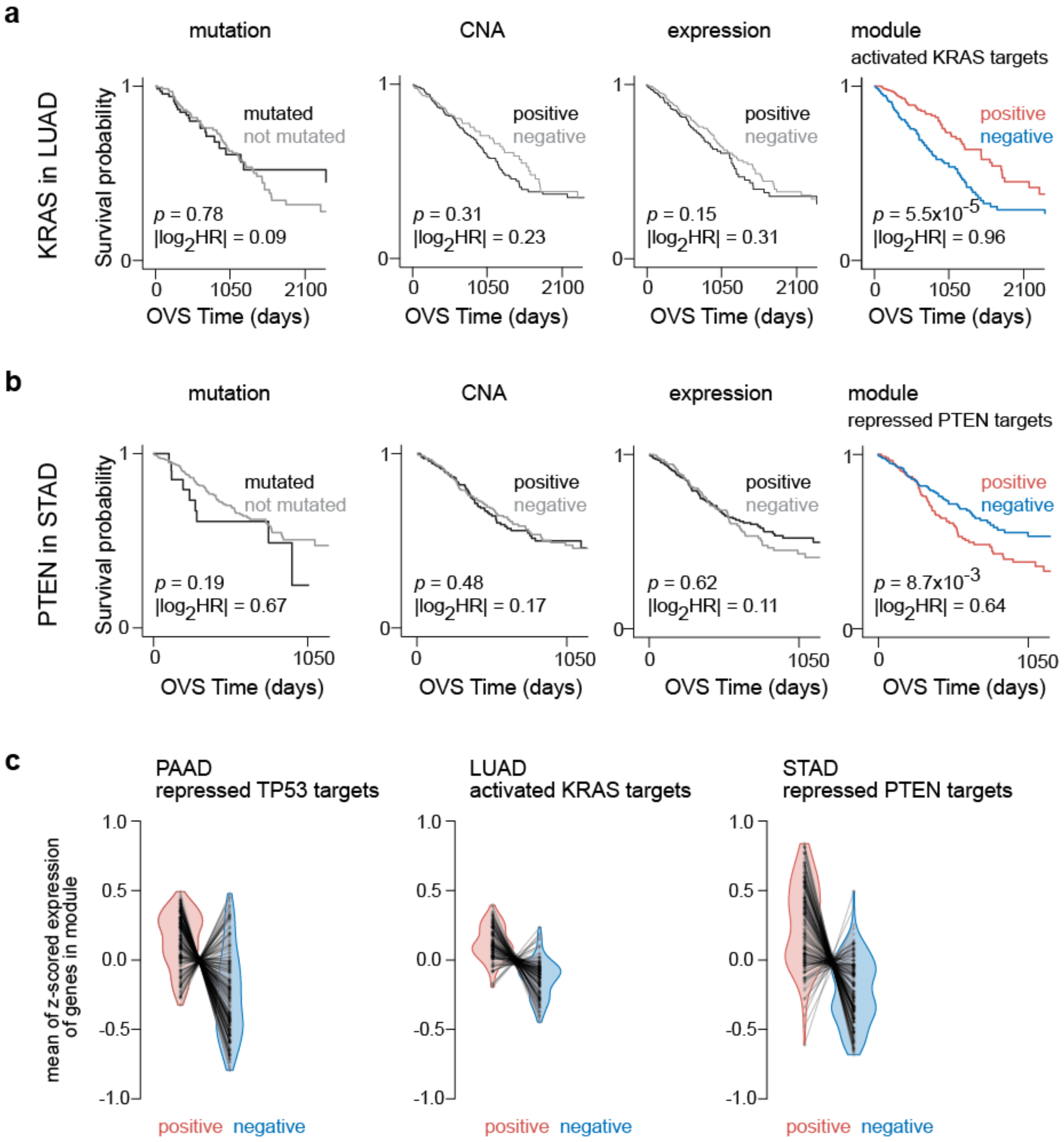
a
mutation
CNA
expression
module
activated KRAS targets
KRAS in LUAD
Survival probability
mutated
not mutated
positive
negative
p = 0.78
|log2HR| = 0.09
p = 0.31
|log2HR| = 0.23
p = 0.15
|log2HR| = 0.31
p = 5.5x10-5
|log2HR| = 0.96
OVS Time (days)
b
mutation
CNA
expression
module
repressed PTEN targets
PTEN in STAD
Survival probability
p = 0.19
|log2HR| = 0.67
p = 0.48
|log2HR| = 0.17
p = 0.62
|log2HR| = 0.11
p = 8.7x10-3
|log2HR| = 0.64
OVS Time (days)
c
PAAD
repressed TP53 targets
LUAD
activated KRAS targets
STAD
repressed PTEN targets
mean of z-scored expression of genes in module
positive
negative

Figure S5. Perturbations in modules associated with KRAS and PTEN provide better patient survival stratification than measurements on individual genes, Related to Figure 1.

(**a**-**b**) Patients stratified based on mutation status, copy-number aberrations and expression changes in (**a**) KRAS in lung adenocarcinoma (LUAD) and, (**b**) PTEN in stomach cancer (STAD). Also shown are patient stratifications based on perturbations in module comprising (**a**) genes up-regulated in lung cancer cells over-expression KRAS (MSigDB[2] M12860) and, (**b**) genes up-regulated upon knockdown of PTEN (MSigDB[2] M2787). (**c**) Violin plots show distributions of the mean expression of genes that make up the modules corresponding to repressed targets of TP53 (MSigDB M2698; 198 genes) in pancreatic cancer (left panel), activated targets of KRAS (MSigDB M12860; 139 genes) in lung adenocarcinoma (middle panel) and repressed targets of PTEN (MSigDB M2787; 186 genes) in stomach cancer cohort (right panel). Patients have been stratified into groups with positive (red) and negative (blue) perturbation scores for these modules. Genes that constitute the modules are shown (black dots) and their mean expression values in the MPS positive and negative groups are linked (black lines). Slopes of the lines indicate whether genes are up- (negative slope) or down-regulated (positive slope) in the MPS positive compared to the MPS negative patient groups. Each module shown comprises of genes with both high and low average expression, while its perturbation scores is statistically significant and it significantly discriminates patients based on survival. Stratification of patients based on the perturbation scores of the three modules shown in this sub-panel are plotted in Fig 1d, Fig S5a (right panel) and Fig S5b (right panel), respectively.

a

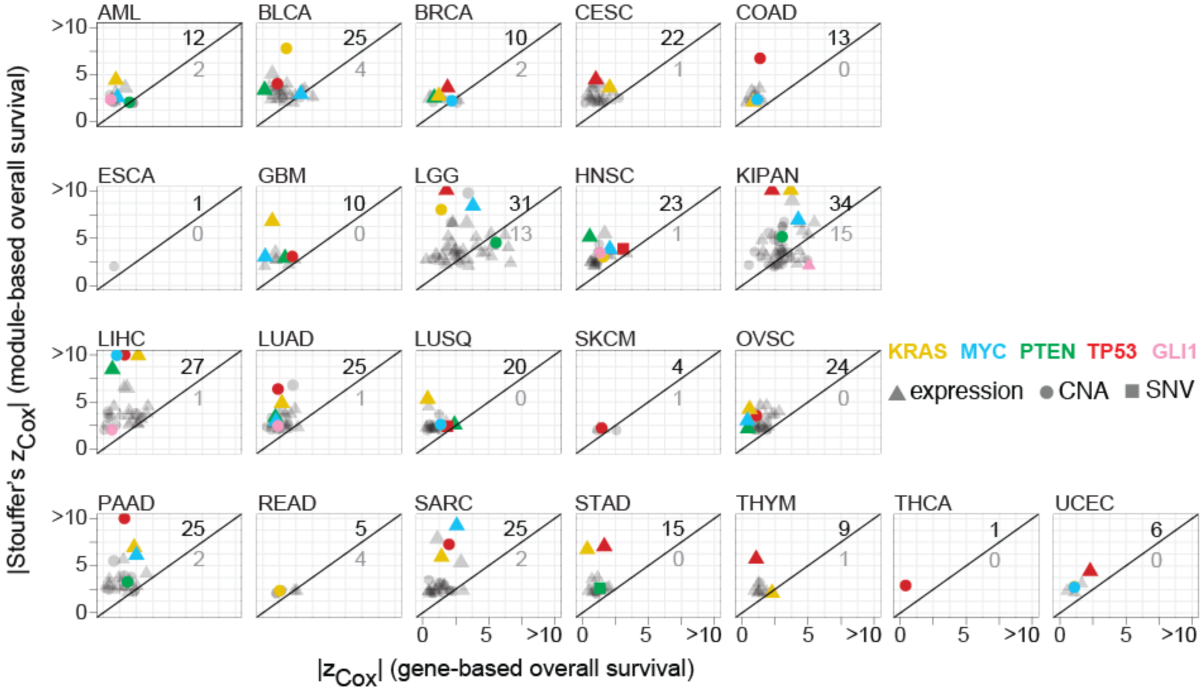

AML BLCA BRCA CESC COAD
ESCA GBM LGG HNSC KIPAN
LIHC LUAD LUSQ SKCM OVSC
PAAD READ SARC STAD THYM THCA UCEC
KRAS MYC PTEN TP53 GLI1
expression CNA SNV
|Stouffer's z_Cox| (module-based overall survival)
|z_Cox| (gene-based overall survival)

b

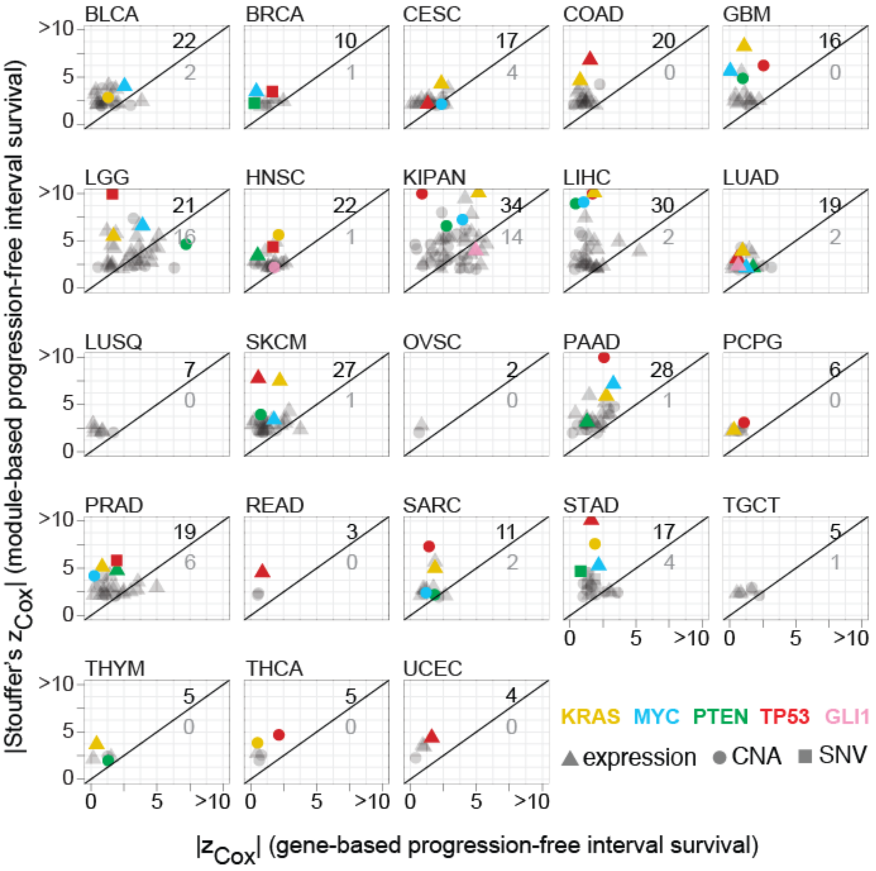

BLCA BRCA CESC COAD GBM
LGG HNSC KIPAN LIHC LUAD
LUSQ SKCM OVSC PAAD PCPG
PRAD READ SARC STAD TGCT
THYM THCA UCEC
KRAS MYC PTEN TP53 GLI1
expression CNA SNV
|Stouffer's z_Cox| (module-based progression-free interval survival)
|z_Cox| (gene-based progression-free interval survival)

Figure S6. Perturbation scores of modules associated with oncogenes and tumor-suppressors provide better patient survival stratification than measurements on individual genes, Related to Figure 1.

(**a**-**b**) Scatterplots show comparisons between absolute standardized significance scores (Wald statistic) individual genes and their corresponding modules (Wald statistic summarized by Stouffer's method) in individual cohorts (labels indicated on top). For individual gene measurements of each oncogene or tumor-suppressor, we report the absolute values of the standardized significance scores for best gene-based stratification (mutation, copy-number or expression; shown in legend). The shape of each dot indicates the nature of measurements used. The modules whose prognostic value is better than the individual gene measurements are above the reference line y=x. The number of oncogenes and tumor-suppressors whose modules have better (worse) prognostic value than individual gene measurements are shown above (below) the reference line. A handful of prominent oncogenes and tumor-suppressors are highlighted (color legend shown). Comparisons are shown for (**a**) overall survival and, (**b**) progression-free interval survival.

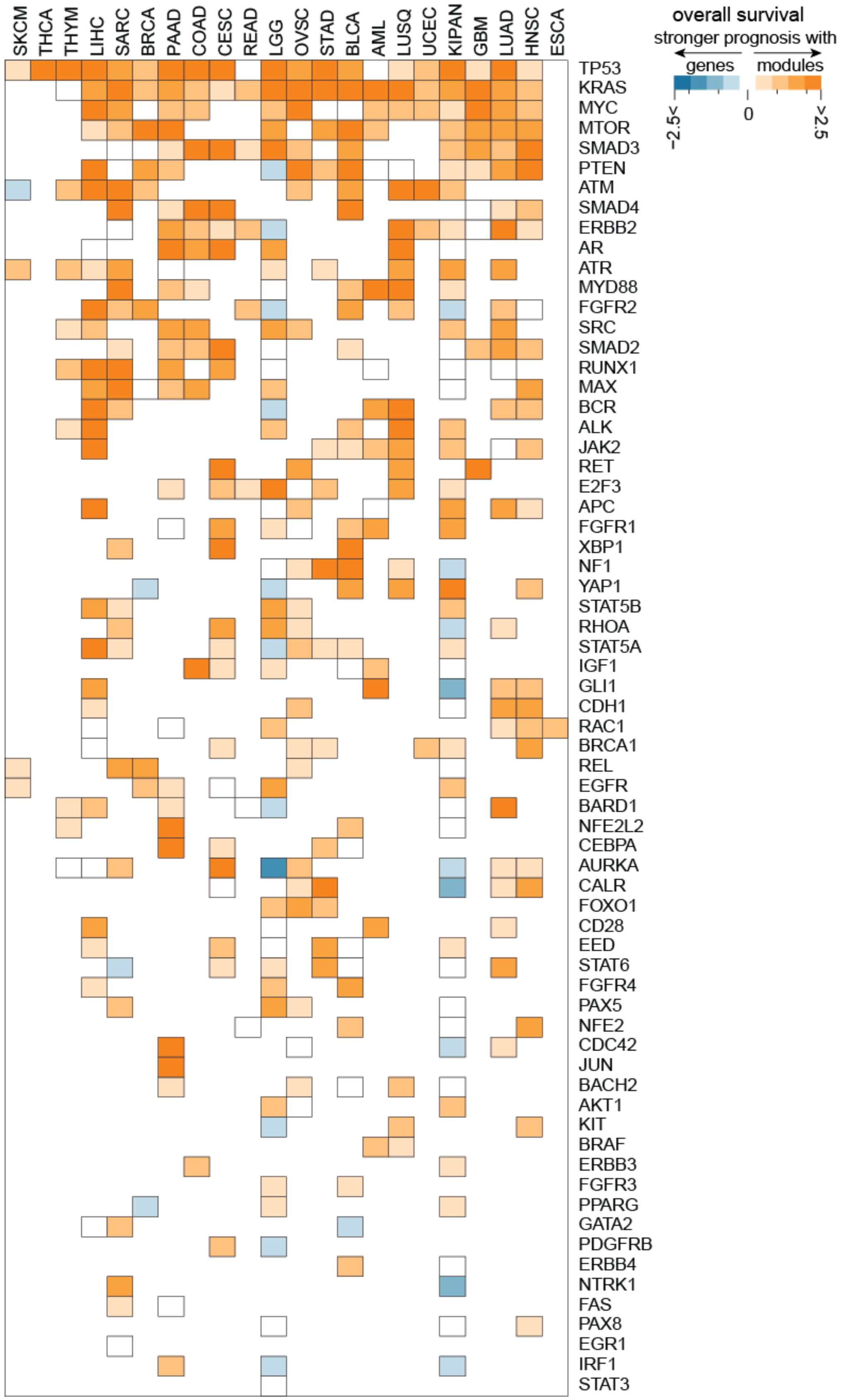
SKCM
THCA
THYM
LIHC
SARC
BRCA
PAAD
COAD
CESC
READ
LGG
OVSC
STAD
BLCA
AML
LUSQ
UCEC
KIPAN
GBM
LUAD
HNSC
ESCA
TP53
KRAS
MYC
MTOR
SMAD3
PTEN
ATM
SMAD4
ERBB2
AR
ATR
MYD88
FGFR2
SRC
SMAD2
RUNX1
MAX
BCR
ALK
JAK2
RET
E2F3
APC
FGFR1
XBP1
NF1
YAP1
STAT5B
RHOA
STAT5A
IGF1
GLI1
CDH1
RAC1
BRCA1
REL
EGFR
BARD1
NFE2L2
CEBPA
AURKA
CALR
FOXO1
CD28
EED
STAT6
FGFR4
PAX5
NFE2
CDC42
JUN
BACH2
AKT1
KIT
BRAF
ERBB3
FGFR3
PPARG
GATA2
PDGFRB
ERBB4
NTRK1
FAS
PAX8
EGR1
IRF1
STAT3
overall survival
stronger prognosis with
genes
modules
−2.5<
0
>2.5

Figure S7. Perturbation scores of modules associated with oncogenes and tumor-suppressors convey stronger overall survival prognosis than measurements on individual genes, Related to Figure 1.

The log_2-ratio of the absolute standardized significance of modules associated with cancer-drivers[1] and measurements on these individual genes (in rows) are visualized across cancers from TCGA (columns). Standardized significance (Wald statistic) for individual genes were chosen to be the maximum from expression, copy-number and mutation based patient stratifications in each cohort. For the corresponding modules, standardized scores were summarized using Stouffer's method (Methods). Rows and columns are clustered using hierarchical clustering using Euclidean distance with optimal ordering of leaves.

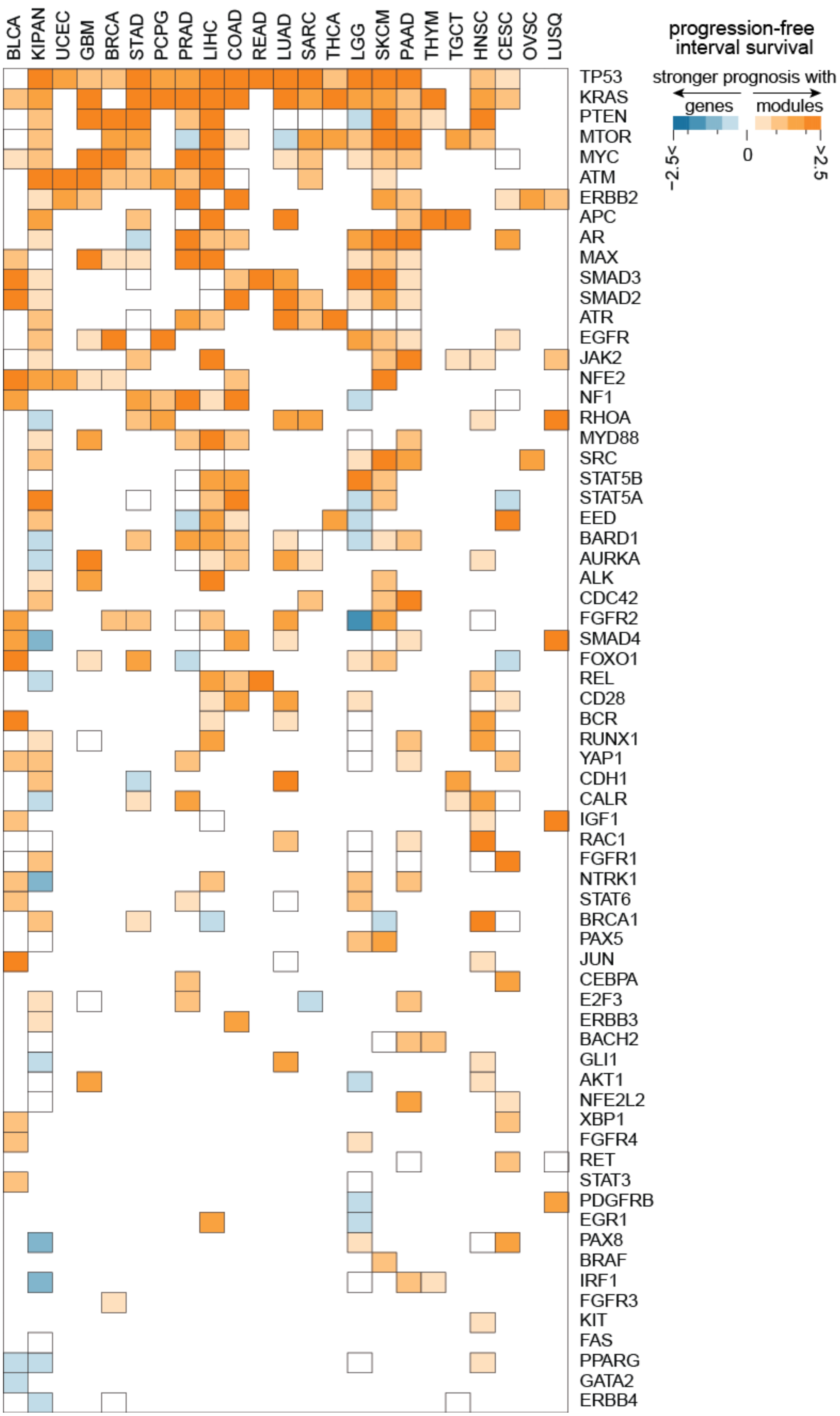
BLCA
KIPAN
UCEC
GBM
BRCA
STAD
PCPG
PRAD
LIHC
COAD
READ
LUAD
SARC
THCA
LGG
SKCM
PAAD
THYM
TGCT
HNSC
CESC
OVSC
LUSQ
TP53
KRAS
PTEN
MTOR
MYC
ATM
ERBB2
APC
AR
MAX
SMAD3
SMAD2
ATR
EGFR
JAK2
NFE2
NF1
RHOA
MYD88
SRC
STAT5B
STAT5A
EED
BARD1
AURKA
ALK
CDC42
FGFR2
SMAD4
FOXO1
REL
CD28
BCR
RUNX1
YAP1
CDH1
CALR
IGF1
RAC1
FGFR1
NTRK1
STAT6
BRCA1
PAX5
JUN
CEBPA
E2F3
ERBB3
BACH2
GLI1
AKT1
NFE2L2
XBP1
FGFR4
RET
STAT3
PDGFRB
EGR1
PAX8
BRAF
IRF1
FGFR3
KIT
FAS
PPARG
GATA2
ERBB4
progression-free interval survival
stronger prognosis with
genes
modules
−2.5<
0
>2.5

Figure S8. Perturbation scores of modules associated with oncogenes and tumor-suppressors convey stronger progression-free interval survival prognosis than measurements on individual genes, Related to Figure 1.

The log_2-ratio of the absolute standardized significance of modules associated with cancer-drivers[1] and measurements on these individual genes (in rows) are visualized across cancers from TCGA (columns). Standardized significance (Wald statistic) for individual genes were chosen to be the maximum from expression, copy-number and mutation based patient stratifications in each cohort. For the corresponding modules, standardized scores were summarized using Stouffer's method (Methods). Rows and columns are clustered using hierarchical clustering using Euclidean distance with optimal ordering of leaves.

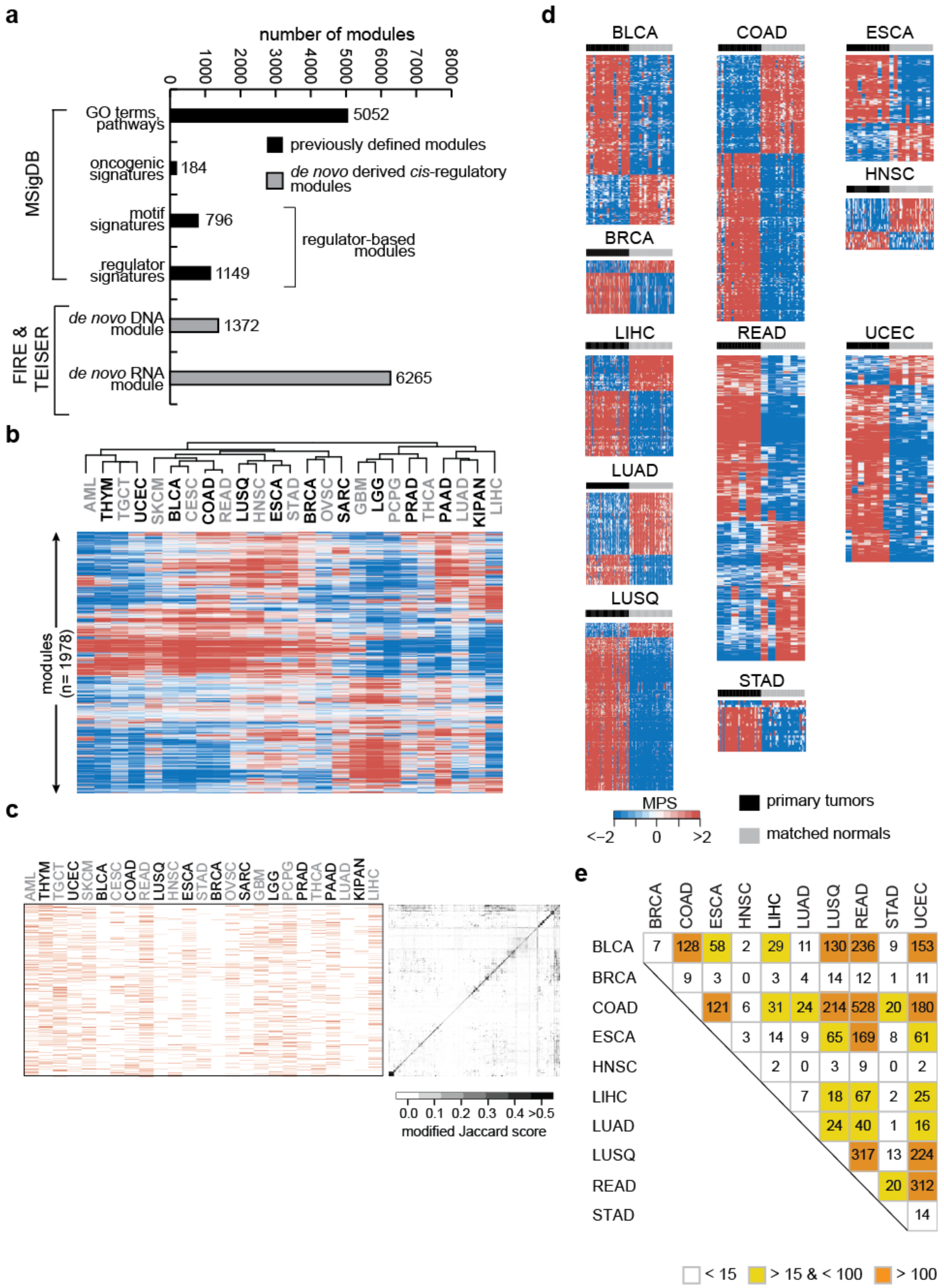
a
number of modules
0
1000
2000
3000
4000
5000
6000
7000
8000
MSigDB
GO terms, pathways
5052
oncogenic signatures
184
motif signatures
796
regulator signatures
1149
regulator-based modules
previously defined modules
de novo derived cis-regulatory modules
FIRE & TEISER
de novo DNA module
1372
de novo RNA module
6265
b
AML THYM TGCT UCEC SKCM BLCA CESC COAD READ LUSQ HNSC ESCA STAD BRCA OVSC SARC GBM LGG PCPG PRAD THCA PAAD LUAD KIPAN LIHC
modules (n= 1978)
c
AML THYM TGCT UCEC SKCM BLCA CESC COAD READ LUSQ HNSC ESCA STAD BRCA OVSC SARC GBM LGG PCPG PRAD THCA PAAD LUAD KIPAN LIHC
0.0 0.1 0.2 0.3 0.4 >0.5
modified Jaccard score
d
BLCA
COAD
ESCA
HNSC
BRCA
LIHC
READ
UCEC
LUAD
LUSQ
STAD
MPS
<-2 0 >2
primary tumors
matched normals
e
BRCA COAD ESCA HNSC LIHC LUAD LUSQ READ STAD UCEC
BLCA 7 128 58 2 29 11 130 236 9 153
BRCA 9 3 0 3 4 14 12 1 11
COAD 121 6 31 24 214 528 20 180
ESCA 3 14 9 65 169 8 61
HNSC 2 0 3 9 0 2
LIHC 7 18 67 2 25
LUAD 24 40 1 16
LUSQ 317 13 224
READ 20 312
STAD 14
< 15
> 15 & < 100
> 100

Figure S9. Module perturbation scores are reflective of cohort-specific states and tumor-normal differences, Related to STAR Methods.

(**a**) Number of all modules considered in this study per category. Previously defined gene modules include well-defined pathways, ontology attributes (from KEGG, PID, REACTOME, Gene Ontology collections), oncogenic signatures (MSigDB) and DNA/RNA sequence motifs characterized to be bound by known regulatory factors[2–5]. Modules associated with *de novo* discovered DNA, linear and structural RNA sequence motifs were discovered using transcriptome analyses of patient biopsies by applying the linear motif discovery algorithm FIRE[6] and the structural motif discovery algorithm TEISER[7]. (**b**) MPS patterns of modules dysregulated in cohort-specific ways (see Methods). Rows (modules) are clustered based on patterns of MPS (scale indicated) in these samples. The median module perturbation scores of cohort-specific modules across samples within each cohort are visualized (scale indicated). (**c**) Heatmaps show the membership of cohort-specific modules in cancers (left panel; Methods section) and the overlap between their constituent genes (right panel). The binary matrix (left panel) indicates modules identified in a cancer in red. The extent of overlap is quantified using a modified Jaccard score (scale indicated; Methods). In both heatmaps, rows (modules) are ordered based on the overlap matrix and columns (cancers) in the binary matrix (left panel) have the same order as in panel (**b**). (**d**) Tumor-specific MPS patterns of modules dysregulated in primary tumors and matched normal samples in 11 different cohorts (labels shown). Rows (modules) are clustered based on patterns of MPS (scale indicated) in these samples. (**e**) Matrix shows number of shared modules dysregulated in primary tumors compared to matched normal samples across 11 different cancer cohorts. The colors indicate the number of shared modules between cancers (color key indicated).

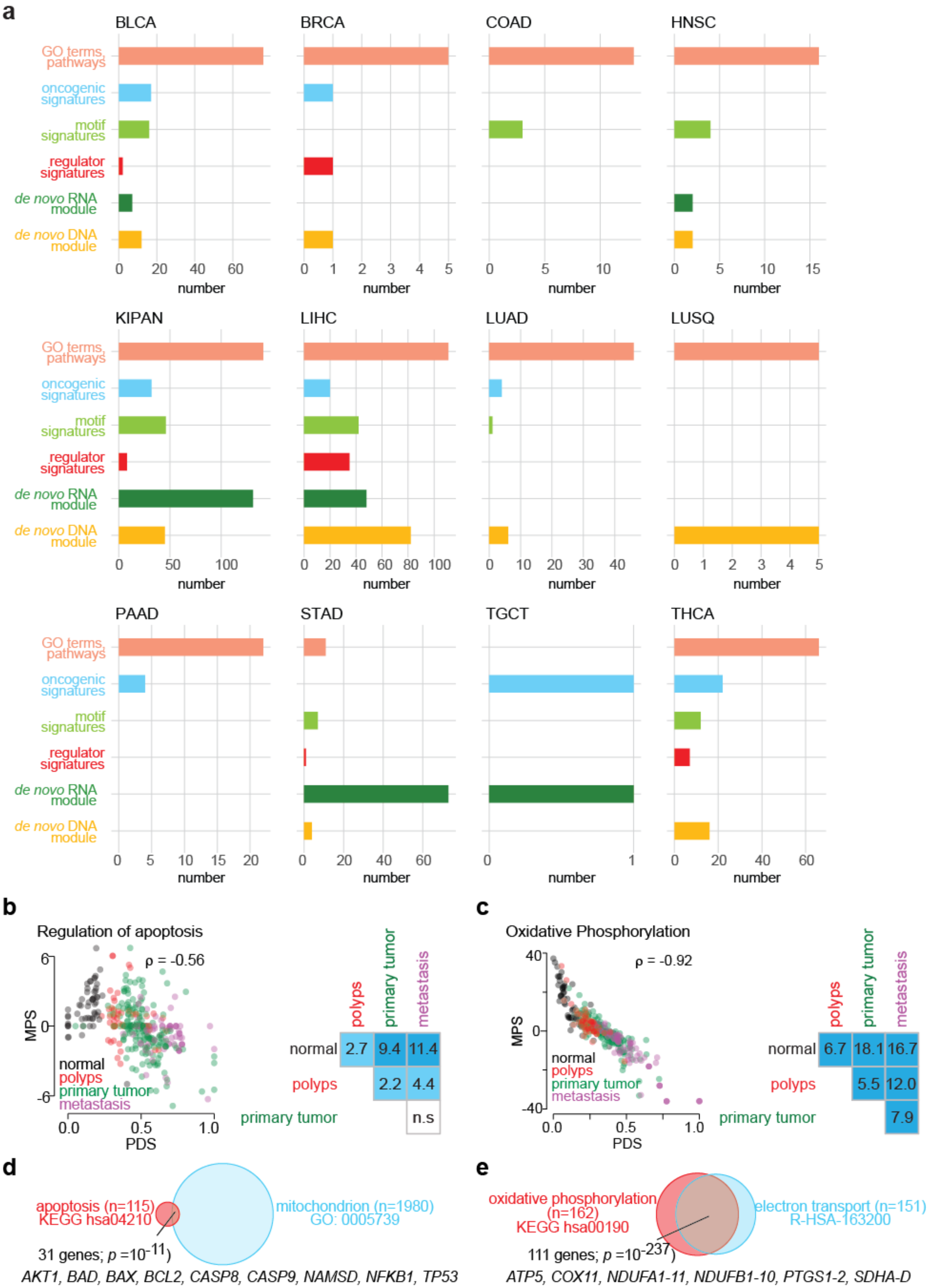
a
BLCA
BRCA
COAD
HNSC
KIPAN
LIHC
LUAD
LUSQ
PAAD
STAD
TGCT
THCA
GO terms, pathways
oncogenic signatures
motif signatures
regulator signatures
de novo RNA module
de novo DNA module
number
b
Regulation of apoptosis
ρ = -0.56
MPS
PDS
normal
polyps
primary tumor
metastasis
normal 2.7 9.4 11.4
polyps 2.2 4.4
primary tumor n.s
c
Oxidative Phosphorylation
ρ = -0.92
normal 6.7 18.1 16.7
polyps 5.5 12.0
primary tumor 7.9
d
apoptosis (n=115)
KEGG hsa04210
mitochondrion (n=1980)
GO: 0005739
31 genes; p =10-11)
AKT1, BAD, BAX, BCL2, CASP8, CASP9, NAMSD, NFKB1, TP53
e
oxidative phosphorylation (n=162)
KEGG hsa00190
electron transport (n=151)
R-HSA-163200
111 genes; p =10-237)
ATP5, COX11, NDUFA1-11, NDUFB1-10, PTGS1-2, SDHA-D

Figure S10. Module perturbation scores are correlated with cancer stage, Related to STAR Methods, Data Sheet 3.

(**a**) Number of modules that are significantly correlated with cancer stage in each module category (Y-axis; module categories indicated) across different TCGA cohorts (labels on top). (**b**-**c**) Scatterplots (left panel) show comparisons between Pathway Deregulation Scores (PDS; X-axis) and Module Perturbation Scores (MPS; Y-axis) for 342 samples obtained from patients with colonic neoplasms (GEO accession GSE41258)[8,9] for modules associated with (**b**) regulation of apoptosis (KEGG database; MSigDB M8492[10]) and (**c**) oxidative phosphorylation (KEGG database; MSigDB M19540[10]). Each dot corresponds to a patient and colors indicate tissue-types (key indicated) and the correlation coefficient between PDS and MPS of these two modules are shown. Significant correlation coefficients between the two measures suggest that that MPS of these modules also capture disease progression just like their PDS, as reported originally by Drier and colleagues[8,11]. We compared the distributions of MPS between the biopsied tissue-types on the independent cohort and the statistics of these comparisons are shown as a heatmap (right panel; $-\log_{10}$ of two-sided Mann-Whitney test *p*-values shown). (**d**) Overlap between apoptosis pathway and module with highest similarity to it (based on hypergeometric test) is shown as a Venn diagram. Statistics of the overlap (hypergeometric test) and some of the prominent overlapping genes are indicated. The module associated with GO term mitochondrion (GO:0005739; 1980 genes) was one of the progression-associated modules discovered in TCGA's colon cancer cohort (panel (**a**)). (**e**) Overlap between oxidative phosphorylation pathway and module with highest similarity to it (based on hypergeometric test) is shown as a Venn diagram. Statistics of the overlap (hypergeometric test) and some of the prominent overlapping genes are indicated. The module associated with electron transport respiratory chain (Reactome R-HSA-163200; 151 genes) was one of the progression-associated modules discovered to be associated with colon cancer stage in TCGA (panel (**a**)).

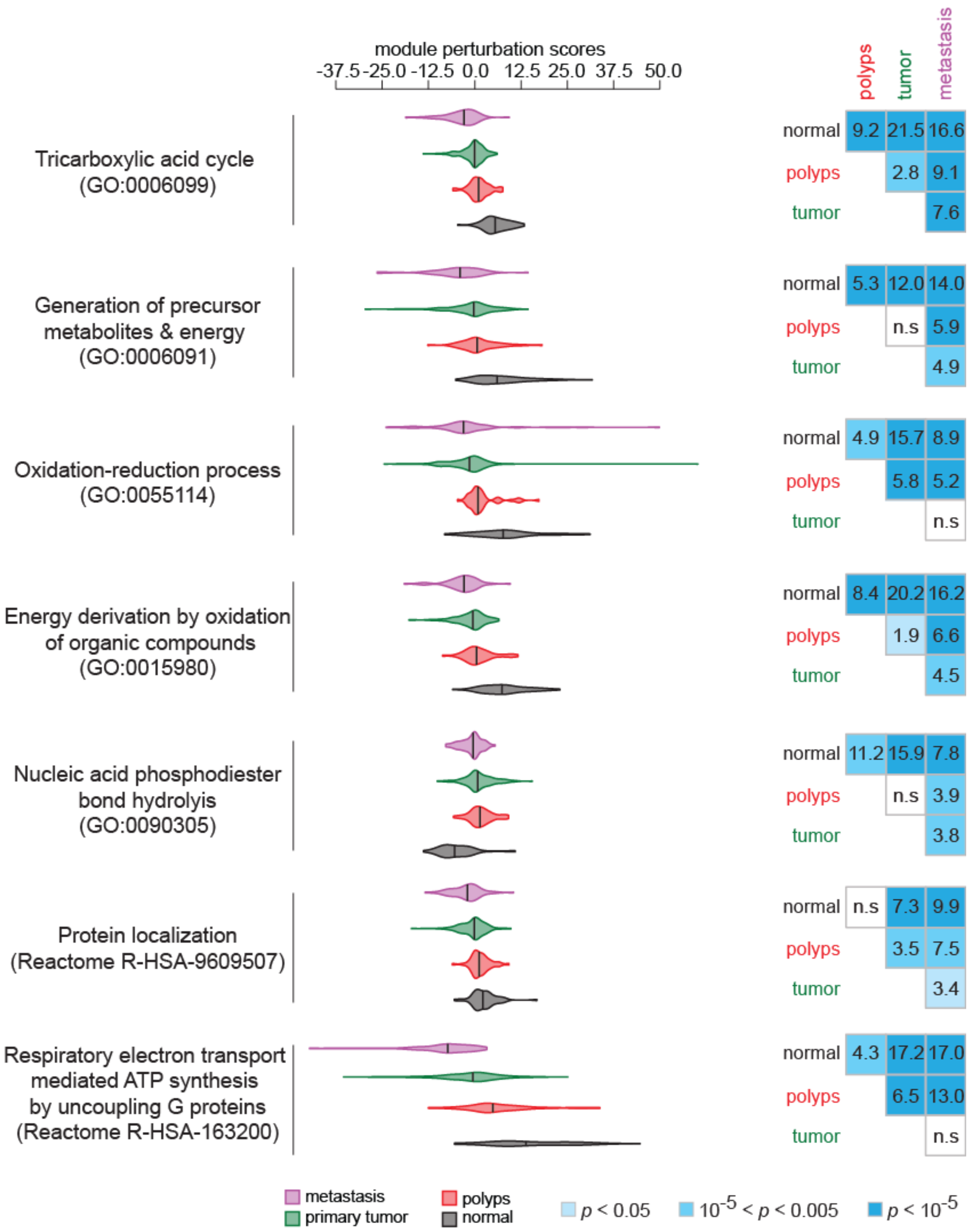
module perturbation scores
-37.5 -25.0 -12.5 0.0 12.5 25.0 37.5 50.0
polyps
tumor
metastasis
Tricarboxylic acid cycle (GO:0006099)
normal 9.2 21.5 16.6
polyps 2.8 9.1
tumor 7.6
Generation of precursor metabolites & energy (GO:0006091)
normal 5.3 12.0 14.0
polyps n.s 5.9
tumor 4.9
Oxidation-reduction process (GO:0055114)
normal 4.9 15.7 8.9
polyps 5.8 5.2
tumor n.s
Energy derivation by oxidation of organic compounds (GO:0015980)
normal 8.4 20.2 16.2
polyps 1.9 6.6
tumor 4.5
Nucleic acid phosphodiester bond hydrolyis (GO:0090305)
normal 11.2 15.9 7.8
polyps n.s 3.9
tumor 3.8
Protein localization (Reactome R-HSA-9609507)
normal n.s 7.3 9.9
polyps 3.5 7.5
tumor 3.4
Respiratory electron transport mediated ATP synthesis by uncoupling G proteins (Reactome R-HSA-163200)
normal 4.3 17.2 17.0
polyps 6.5 13.0
tumor n.s
metastasis
primary tumor
polyps
normal
p < 0.05
10-5 < p < 0.005
p < 10-5

Figure S11. Modules discovered on TCGA's colon cancer cohort are correlated with disease progression on an independent colon cancer cohort, Related to STAR Methods, Data Sheet 3.

For progression-associated modules discovered on TCGA's colon cancer cohort, the distributions of perturbation scores (modules indicated) in metastatic, primary tumor, polyps and normal tissues biopsied from patients with colonic neoplasms (GEO accession GSE41258)[8,9] are shown as violin plots (left panel). For each module, heatmap (right panel) shows statistics of the comparisons between MPS distributions in the biopsied tissue-types (right panel; two-sided Mann-Whitney test *p*-values shown).

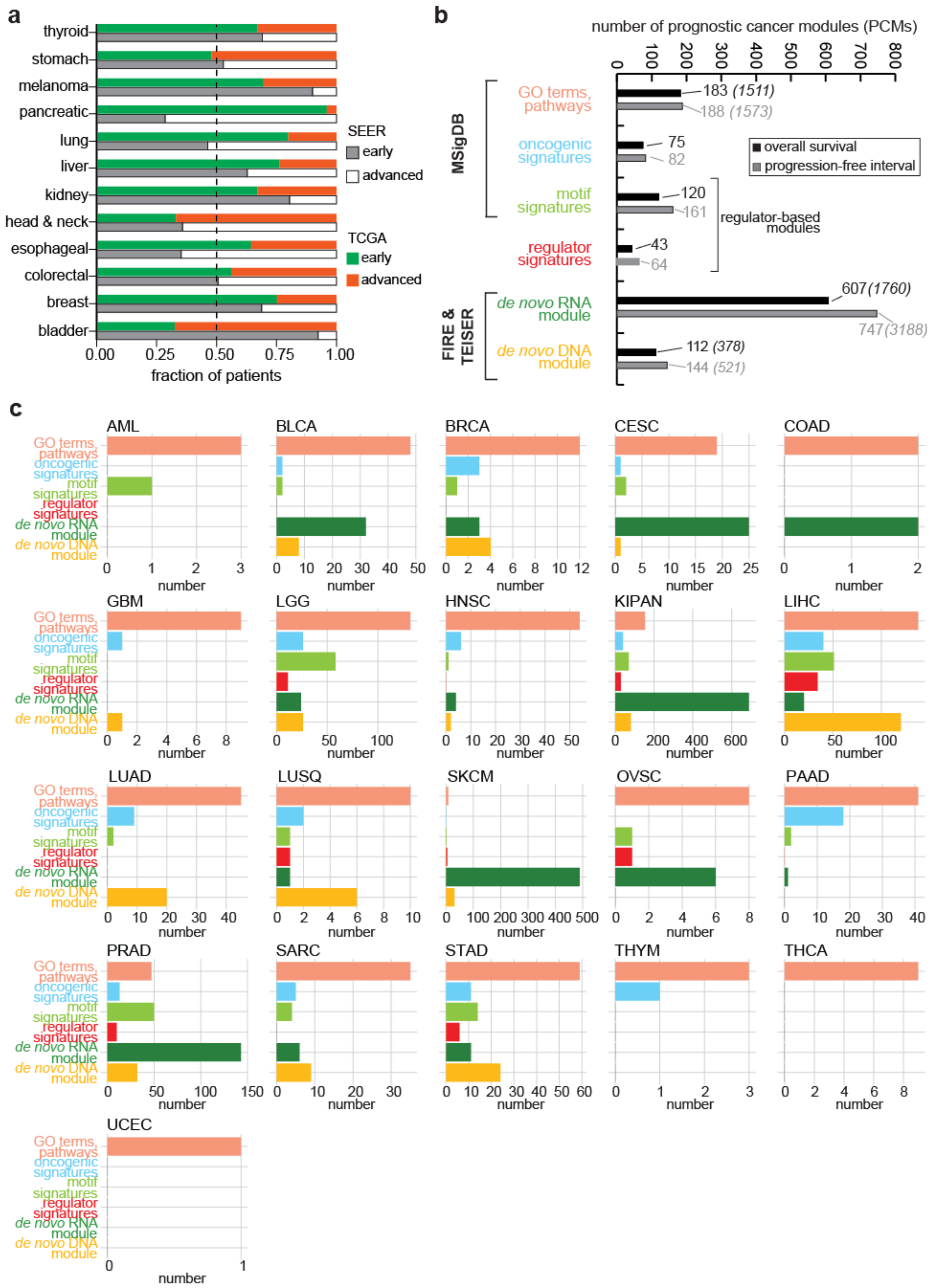
a
thyroid
stomach
melanoma
pancreatic
lung
liver
kidney
head & neck
esophageal
colorectal
breast
bladder
0.00
0.25
0.50
0.75
1.00
fraction of patients
SEER
early
advanced
TCGA
early
advanced
b
number of prognostic cancer modules (PCMs)
MSigDB
GO terms, pathways
oncogenic signatures
motif signatures
regulator signatures
FIRE & TEISER
de novo RNA module
de novo DNA module
183 (1511)
188 (1573)
75
82
120
161
43
64
607 (1760)
747 (3188)
112 (378)
144 (521)
overall survival
progression-free interval
regulator-based modules
c
AML
BLCA
BRCA
CESC
COAD
GBM
LGG
HNSC
KIPAN
LIHC
LUAD
LUSQ
SKCM
OVSC
PAAD
PRAD
SARC
STAD
THYM
THCA
UCEC
number

Figure S12. Numbers of prognostic cancer modules across different module categories for different cancers, Related to STAR Methods, Data Sheet 4.

(a) Fraction of patients with early or advanced stage cancer at time of diagnosis from the SEER database and in tissue-matched TCGA cohorts (Methods). Cancers are shown in rows. **(b)** Number of modules in each module category that specify significant prognostic value for overall survival (black bars) and progression-free interval survival (gray bars). For GO terms and pathways, *de novo* discovered DNA and RNA modules, numbers refer to non-redundant modules (as defined through Affinity Propagation[12]) and the numbers in parentheses, the full set of redundant modules (see Methods). (**c**) Number of prognostic cancer modules (both overall survival and progression-free interval survival) in each module category (Y-axis; module categories indicated) across different TCGA cohorts (labels on top).

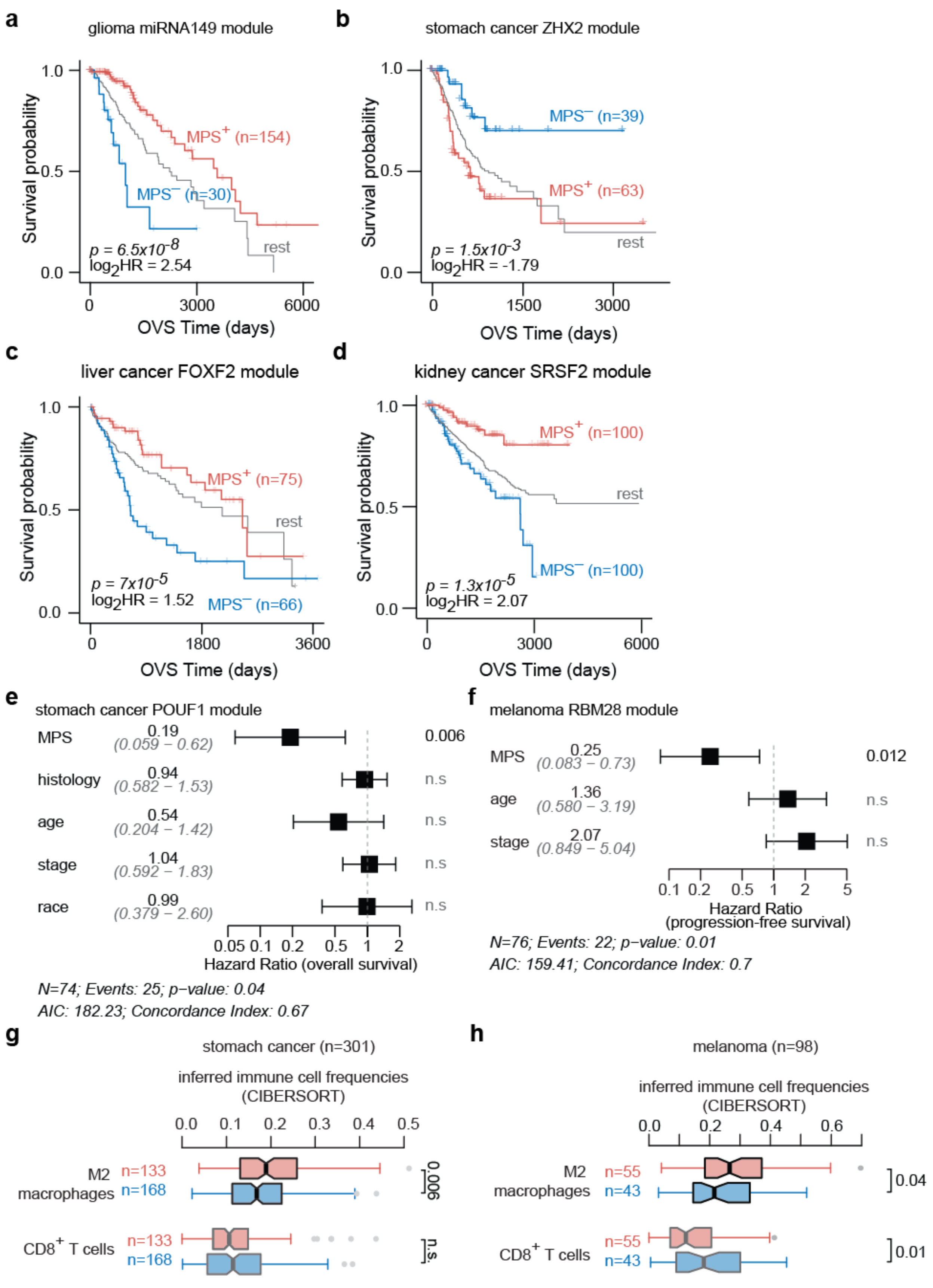

a
glioma miRNA149 module
Survival probability
1.0
0.5
0.0
MPS+ (n=154)
MPS− (n=30)
rest
p = 6.5x10-8
log2HR = 2.54
0
3000
6000
OVS Time (days)
b
stomach cancer ZHX2 module
MPS− (n=39)
MPS+ (n=63)
rest
p = 1.5x10-3
log2HR = -1.79
0
1500
3000
OVS Time (days)
c
liver cancer FOXF2 module
MPS+ (n=75)
rest
MPS− (n=66)
p = 7x10-5
log2HR = 1.52
0
1800
3600
OVS Time (days)
d
kidney cancer SRSF2 module
MPS+ (n=100)
rest
MPS− (n=100)
p = 1.3x10-5
log2HR = 2.07
0
3000
6000
OVS Time (days)
e
stomach cancer POUF1 module
MPS 0.19 (0.059 – 0.62) 0.006
histology 0.94 (0.582 – 1.53) n.s
age 0.54 (0.204 – 1.42) n.s
stage 1.04 (0.592 – 1.83) n.s
race 0.99 (0.379 – 2.60) n.s
0.05 0.1 0.2 0.5 1 2
Hazard Ratio (overall survival)
N=74; Events: 25; p−value: 0.04
AIC: 182.23; Concordance Index: 0.67
f
melanoma RBM28 module
MPS 0.25 (0.083 – 0.73) 0.012
age 1.36 (0.580 – 3.19) n.s
stage 2.07 (0.849 – 5.04) n.s
0.1 0.2 0.5 1 2 5
Hazard Ratio
(progression-free survival)
N=76; Events: 22; p−value: 0.01
AIC: 159.41; Concordance Index: 0.7
g
stomach cancer (n=301)
inferred immune cell frequencies
(CIBERSORT)
0.0 0.1 0.2 0.3 0.4 0.5
M2 macrophages n=133 n=168
0.006
CD8+ T cells n=133 n=168
n.s.
h
melanoma (n=98)
inferred immune cell frequencies
(CIBERSORT)
0.0 0.2 0.4 0.6
M2 macrophages n=55 n=43
0.04
CD8+ T cells n=55 n=43
0.01

Figure S13. Patient survival stratification using previously defined modules associated with transcription factors, miRNAs and RNA binding proteins, Related to Figure 2, Data Sheet 4.

(**a**) Glioma patients with significant module activation (MPS^{+}; red) for transcripts carrying at least one instance of the motif GAGCCAG in their 3'UTRs (miRNA-149 module; MSigDB[2] M2014; 149 genes) showed better overall survival (OVS) prognosis compared to samples with significant module repression (MPS^{-}; blue). (**b**) Stomach cancer patients with significant module activation (MPS^{+}; red) for genes carrying at least one instance of ZHX2 binding sites in their promoters (MSigDB[2] M14351; 271 genes) showed worse overall survival (OVS) prognosis compared to samples with significant module repression (MPS^{-}; blue). (**c**) Liver cancer patients with significant module activation (MPS^{+}; red) for genes carrying at least one instance of FOXF2 binding sites in their promoters (MSigDB[2] M3746; 939 genes) showed better overall survival (OVS) prognosis compared to samples with significant module repression (MPS^{-}; blue). (**d**) Kidney cancer patients with significant module activation (MPS^{+}; red) for genes carrying at least one instance of SRSF2 binding sites (CISBP-RNA[4]; 671 genes) showed better overall survival (OVS) prognosis compared to samples with significant module repression (MPS^{-}; blue). (**e**) Forest plot for stomach cancer patients with a module corresponding to genes carrying at least one instance of the binding site for POU1F1. Shown are results from multivariate Cox analysis incorporating module perturbation scores (MPS), histological types, age, stage and race. For overall survival prognosis, hazard ratios (horizontal axis) with 95% confidence intervals and *p*-values are shown for each variable. (**f**) Forest plot for melanoma patients with a module corresponding to genes carrying at least one instance of RBM28 binding sites in their 3'UTRs. Shown are results from multivariate cox analysis incorporating module perturbation scores (MPS), age and stage. For progression-free interval survival prognosis, hazard ratios (horizontal axis) with 95% confidence intervals and *p*-values are shown for each variable. (**g**) Boxplots show the distributions of inferred frequencies of M2 Macrophages and CD8^{+} T cells (CIBERSORT[13]) in stomach cancer patients with significant activation (red) or repression (blue) of the module associated with POU1F1. One-sided Mann-Whitney test *p*-values are indicated. (**h**) Boxplots show the distributions of inferred frequencies of M2 Macrophages and CD8^{+} T cells (CIBERSORT[13]) in melanoma patients with significant activation (red) or repression (blue) of the module associated with RBM28. One-sided Mann-Whitney test *p*-values are indicated. For all KM plot comparisons, statistics (median survival times, $\log_2$ hazard ratio and *p*-value) are indicated and survival of the rest of the samples is shown in gray.

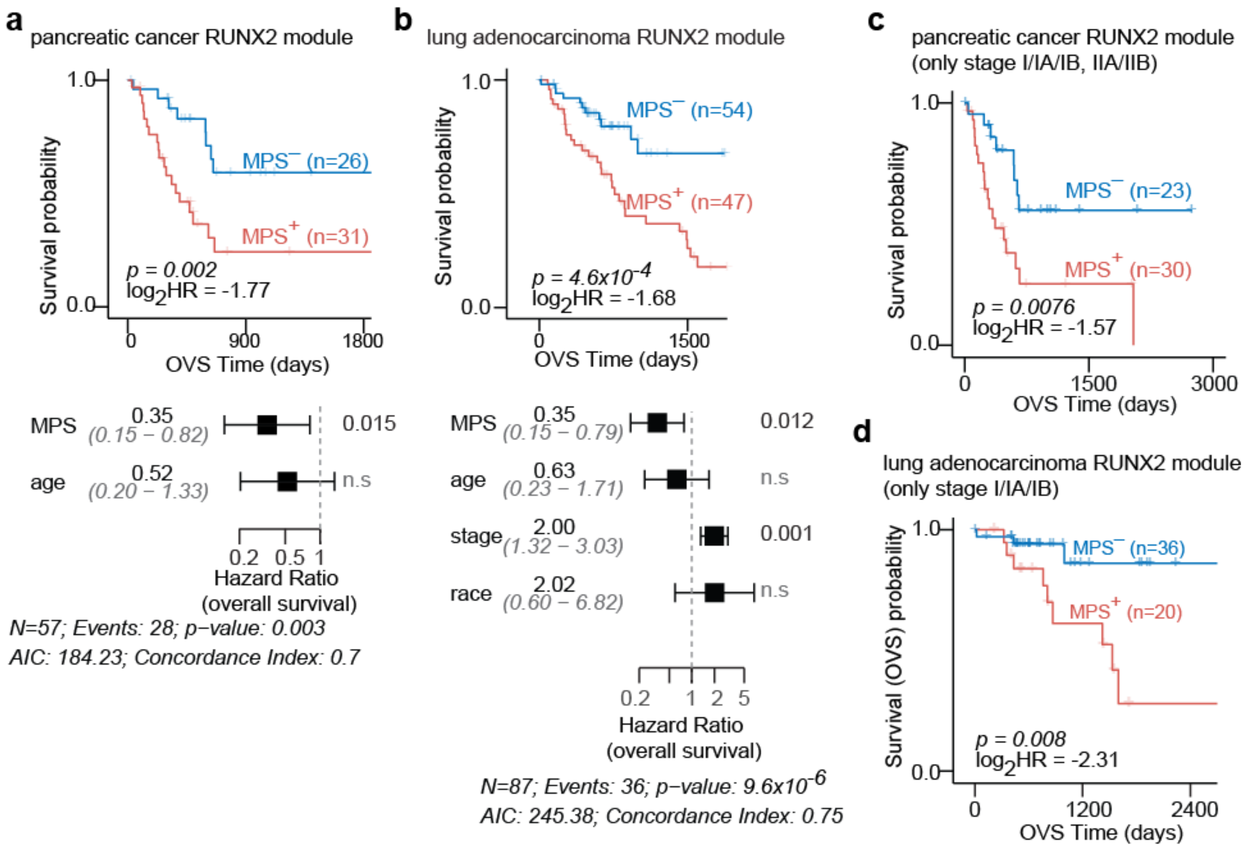

a
pancreatic cancer RUNX2 module
Survival probability
1.0
0.0
MPS⁻ (n=26)
MPS⁺ (n=31)
p = 0.002
log2HR = -1.77
0
900
1800
OVS Time (days)
MPS 0.35 (0.15 – 0.82) 0.015
age 0.52 (0.20 – 1.33) n.s
0.2 0.5 1
Hazard Ratio
(overall survival)
N=57; Events: 28; p-value: 0.003
AIC: 184.23; Concordance Index: 0.7
b
lung adenocarcinoma RUNX2 module
Survival probability
1.0
0.0
MPS⁻ (n=54)
MPS⁺ (n=47)
p = 4.6x10-4
log2HR = -1.68
0
1500
OVS Time (days)
MPS 0.35 (0.15 – 0.79) 0.012
age 0.63 (0.23 – 1.71) n.s
stage 2.00 (1.32 – 3.03) 0.001
race 2.02 (0.60 – 6.82) n.s
0.2 1 2 5
Hazard Ratio
(overall survival)
N=87; Events: 36; p-value: 9.6x10-6
AIC: 245.38; Concordance Index: 0.75
c
pancreatic cancer RUNX2 module
(only stage I/IA/IB, IIA/IIB)
Survival probability
1.0
0.0
MPS⁻ (n=23)
MPS⁺ (n=30)
p = 0.0076
log2HR = -1.57
0
1500
3000
OVS Time (days)
d
lung adenocarcinoma RUNX2 module
(only stage I/IA/IB)
Survival (OVS) probability
1.0
0.0
MPS⁻ (n=36)
MPS⁺ (n=20)
p = 0.008
log2HR = -2.31
0
1200
2400
OVS Time (days)

Figure S14. RUNX2 associated module conveys clinical significance in lung and pancreatic cancers, Related to Figure 2.

(**a**) Patients with significant module activation (MPS^+; red) for genes that are transcriptional targets of RUNX2 (MSigDB[2] M27785; 125 genes) have worse overall survival (OVS) than patients with significant module repression ($MPS^–$; blue) in pancreatic cancer patients (top panel). Forest plot shows results from multivariate cox analysis incorporating module perturbation scores (MPS) and age (bottom panel). (**b**) Patients with significant module activation (MPS^+; red) for genes that are transcriptional targets of RUNX2 (MSigDB[2] M27785; 125 genes) have worse overall survival (OVS) than patients with significant module repression ($MPS^–$; blue) in lung adenocarcinoma patients (top panel). Forest plot shows results from multivariate cox analysis incorporating module perturbation scores (MPS) and age (bottom panel). For visualization, time axes of KM curves are trimmed when the percentage of samples in MPS^+ or $MPS^–$ groups fall below 5%. In forest plots, hazard ratios (horizontal axis) with 95% confidence intervals and *p*-values are shown for each variable. (**c**-**d**) For the module associated with RUNX2, MPS-based patient stratification in specified sub-cohorts to only include early-stage tumors in (**c**) pancreatic cancer and, (**d**) lung adenocarcinoma patients. Module activation (MPS^+; red) was associated with worse overall survival outcomes than module repression ($MPS^–$; blue). Statistics of the comparisons are indicated. For all KM plot comparisons, statistics (median survival times, $\log_2$ hazard ratio and *p*-value) are indicated.

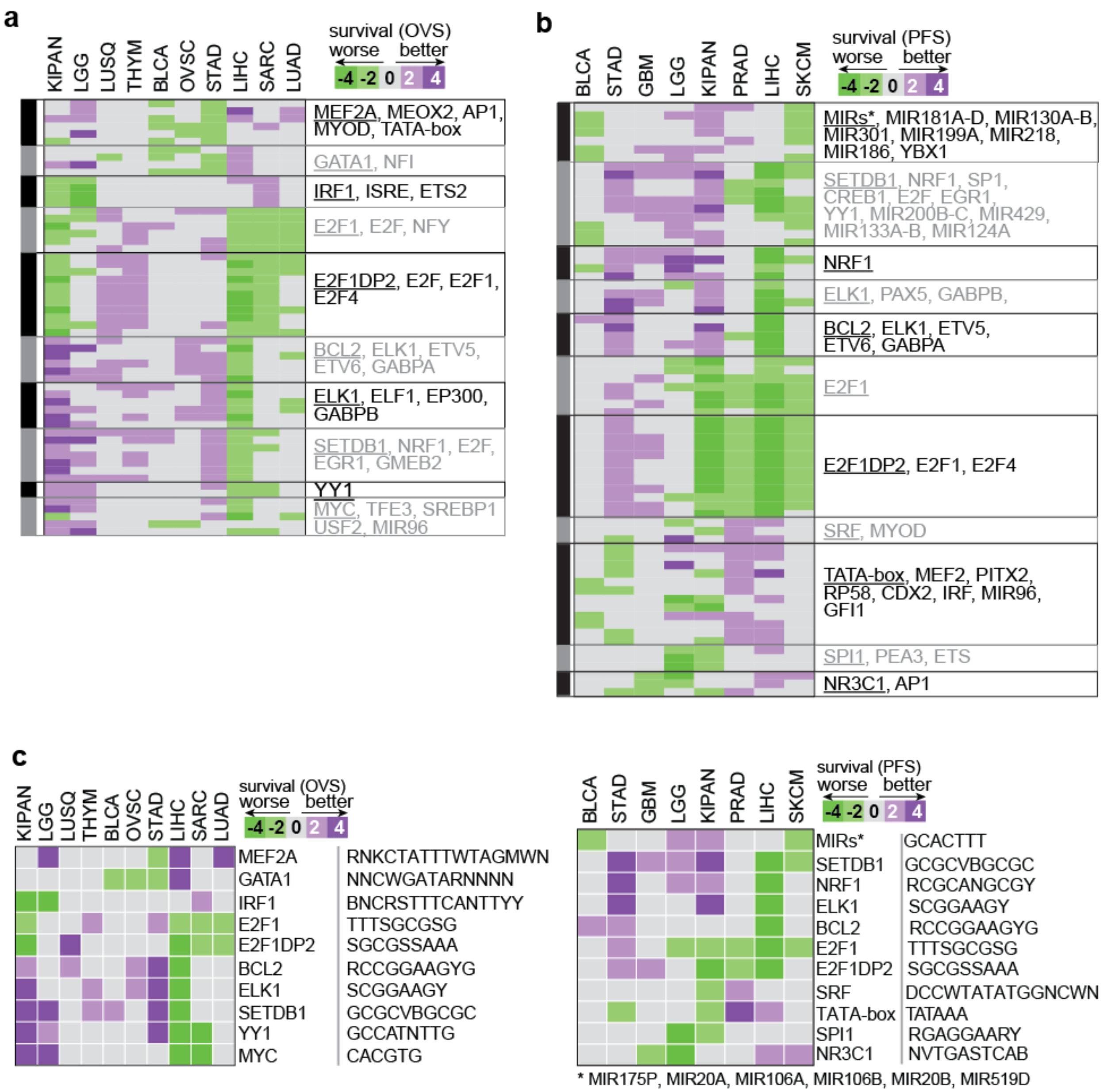
a
KIPAN LGG LUSQ THYM BLCA OVSC STAD LIHC SARC LUAD
survival (OVS)
worse better
-4 -2 0 2 4
MEF2A, MEOX2, AP1, MYOD, TATA-box
GATA1, NFI
IRF1, ISRE, ETS2
E2F1, E2F, NFY
E2F1DP2, E2F, E2F1, E2F4
BCL2, ELK1, ETV5, ETV6, GABPA
ELK1, ELF1, EP300, GABPB
SETDB1, NRF1, E2F, EGR1, GMEB2
YY1
MYC, TFE3, SREBP1 USF2, MIR96
b
BLCA STAD GBM LGG KIPAN PRAD LIHC SKCM
survival (PFS)
worse better
-4 -2 0 2 4
MIRs*, MIR181A-D, MIR130A-B, MIR301, MIR199A, MIR218, MIR186, YBX1
SETDB1, NRF1, SP1, CREB1, E2F, EGR1, YY1, MIR200B-C, MIR429, MIR133A-B, MIR124A
NRF1
ELK1, PAX5, GABPB,
BCL2, ELK1, ETV5, ETV6, GABPA
E2F1
E2F1DP2, E2F1, E2F4
SRF, MYOD
TATA-box, MEF2, PITX2, RP58, CDX2, IRF, MIR96, GFI1
SPI1, PEA3, ETS
NR3C1, AP1
c
KIPAN LGG LUSQ THYM BLCA OVSC STAD LIHC SARC LUAD
survival (OVS)
worse better
-4 -2 0 2 4
MEF2A RNKCTATTTWTAGMWN
GATA1 NNCWGATARNNNN
IRF1 BNCRSTTTCANTTYY
E2F1 TTTSGCGSG
E2F1DP2 SGCGSSAAA
BCL2 RCCGGAAGYG
ELK1 SCGGAAGY
SETDB1 GCGCVBGCGC
YY1 GCCATNTTG
MYC CACGTG
BLCA STAD GBM LGG KIPAN PRAD LIHC SKCM
survival (PFS)
worse better
-4 -2 0 2 4
MIRs* GCACTTT
SETDB1 GCGCVBGCGC
NRF1 RCGCANGCGY
ELK1 SCGGAAGY
BCL2 RCCGGAAGYG
E2F1 TTTSGCGSG
E2F1DP2 SGCGSSAAA
SRF DCCWTATATGGNCWN
TATA-box TATAAA
SPI1 RGAGGAARY
NR3C1 NVTGASTCAB
* MIR175P, MIR20A, MIR106A, MIR106B, MIR20B, MIR519D

Figure S15. Identifying regulator-based modules conveying clinical significance in multiple cancers, Related to Figure 2.

(**a**-**b**) Standardized significance of MPS based patient survival (Wald statistic) of recurrently prognostic regulator-based modules (rows) shown across cancer cohorts (columns) for (**a**) overall survival (OVS) and (**b**) progression-free interval survival (PFS). Positive (or negative) values indicate worse (or better) survival of patients with significant module activation compared to repression. For each module in every cohort, samples were stratified into MPS^+ and MPS^- groups to quantify survival differences between patients in the two groups. Stratifications that result in significant survival differences (KM test *p*-value < 0.05) are retained. Recurrently prognostic regulator-based PCMs are clustered using Affinity Propagation[12] to identify groups of modules that share substantial overlap between them. Similarities between modules were quantified using a modified Jaccard score (Methods). Rows are grouped together based on the clusters identified (indicated) and regulators associated with the exemplar module within each cluster are indicated. (**c**) Standardized significance of MPS based patient survival (Wald statistic) of exemplar regulator-associated modules (rows; motif IUPAC symbols indicated) specifying significant overall survival (OVS; top panel) and progression-free interval survival (PFS; bottom panel) across TCGA cohorts (columns).

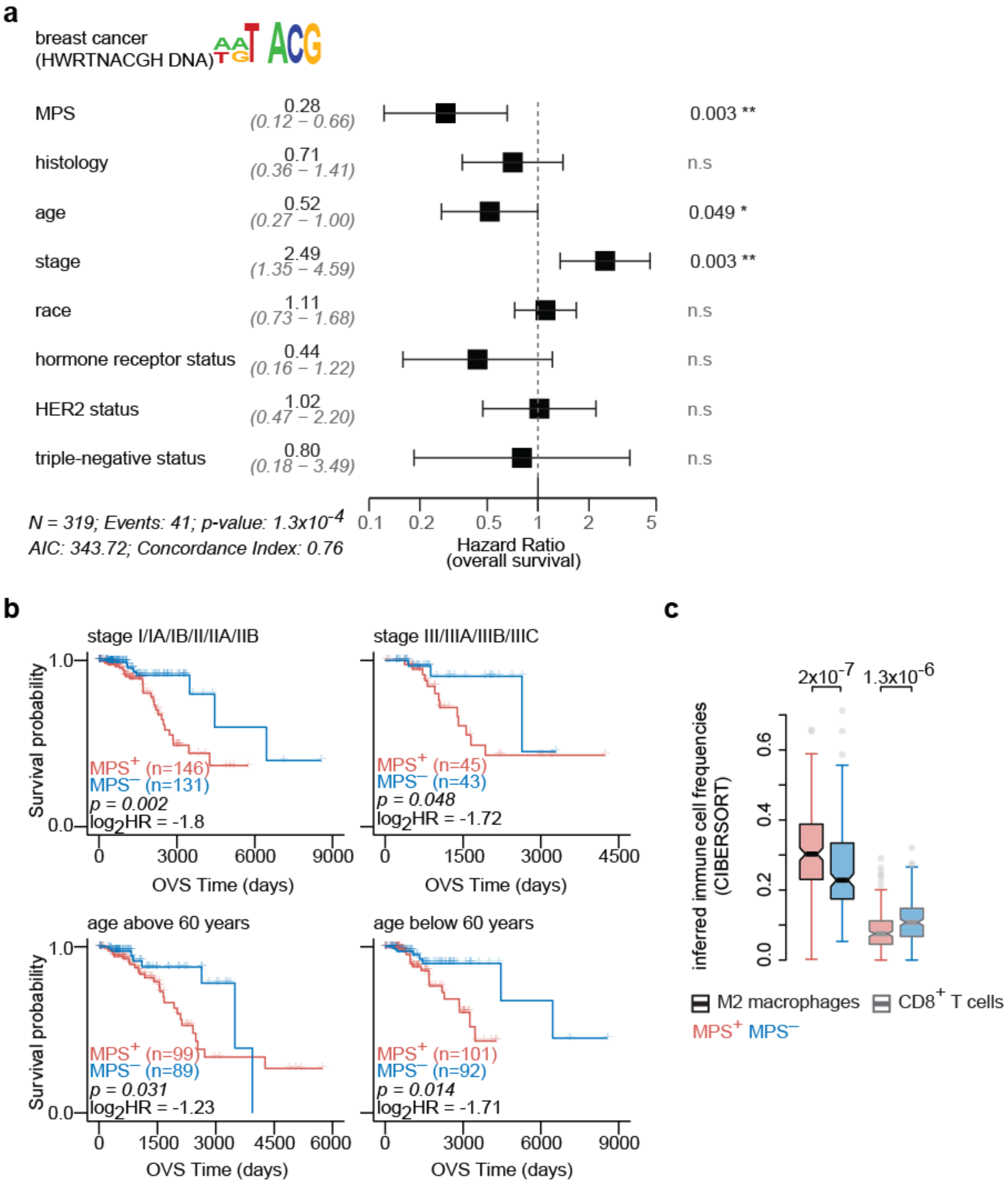

a
breast cancer
(HWRTNACGH DNA)
MPS
0.28
(0.12 – 0.66)
0.003 **
histology
0.71
(0.36 – 1.41)
n.s
age
0.52
(0.27 – 1.00)
0.049 *
stage
2.49
(1.35 – 4.59)
0.003 **
race
1.11
(0.73 – 1.68)
n.s
hormone receptor status
0.44
(0.16 – 1.22)
n.s
HER2 status
1.02
(0.47 – 2.20)
n.s
triple-negative status
0.80
(0.18 – 3.49)
n.s
N = 319; Events: 41; p-value: 1.3x10-4
AIC: 343.72; Concordance Index: 0.76
Hazard Ratio
(overall survival)
b
stage I/IA/IB/II/IIA/IIB
MPS+ (n=146)
MPS− (n=131)
p = 0.002
log2HR = -1.8
stage III/IIIA/IIIB/IIIC
MPS+ (n=45)
MPS− (n=43)
p = 0.048
log2HR = -1.72
age above 60 years
MPS+ (n=99)
MPS− (n=89)
p = 0.031
log2HR = -1.23
age below 60 years
MPS+ (n=101)
MPS− (n=92)
p = 0.014
log2HR = -1.71
Survival probability
OVS Time (days)
c
2x10-7
1.3x10-6
inferred immune cell frequencies (CIBERSORT)
M2 macrophages
CD8+ T cells
MPS+ MPS−

Figure S16. A *de novo* discovered DNA-based *cis*-regulatory module specifies significant prognosis in breast cancer even after controlling for confounds, Related to Figure 3.

(**a**) Forest plot for breast cancer patients with a *de novo* discovered, *cis*-regulatory prognostic cancer module corresponding to transcripts carrying at least one instance of the DNA motif HWRTNACGH (logo shown) within the first 1KB of their promoters. Shown are results from multivariate cox analysis incorporating module perturbation scores (MPS), histological type, age, stage, race, hormone receptor status, HER2 status and triple-negative status. (**b**) MPS-based patient stratification for this PCM (previous panel) in specified sub-cohorts of the breast cancer patients including stage I and II patients (top; left panel), stage III patients (top; right panel), patients whose age is above 60 years at time of diagnosis (bottom; left panel) and patients whose age is less than 60 years at time of diagnosis (bottom; right panel). Module activation (MPS^+; red) was associated with worse overall survival outcomes than module repression ($MPS^–$; blue). (**c**) Boxplots show the inferred frequencies of M2 Macrophages and $CD8^+$ T cells (CIBERSORT[13]) in breast cancer patients with significant activation (MPS^+; red) or repression ($MPS^–$; blue) of the module associated with the DNA motif HWRTNACGH (module in (**a**-**b**)). One-sided Mann-Whitney test *p*-values are indicated.

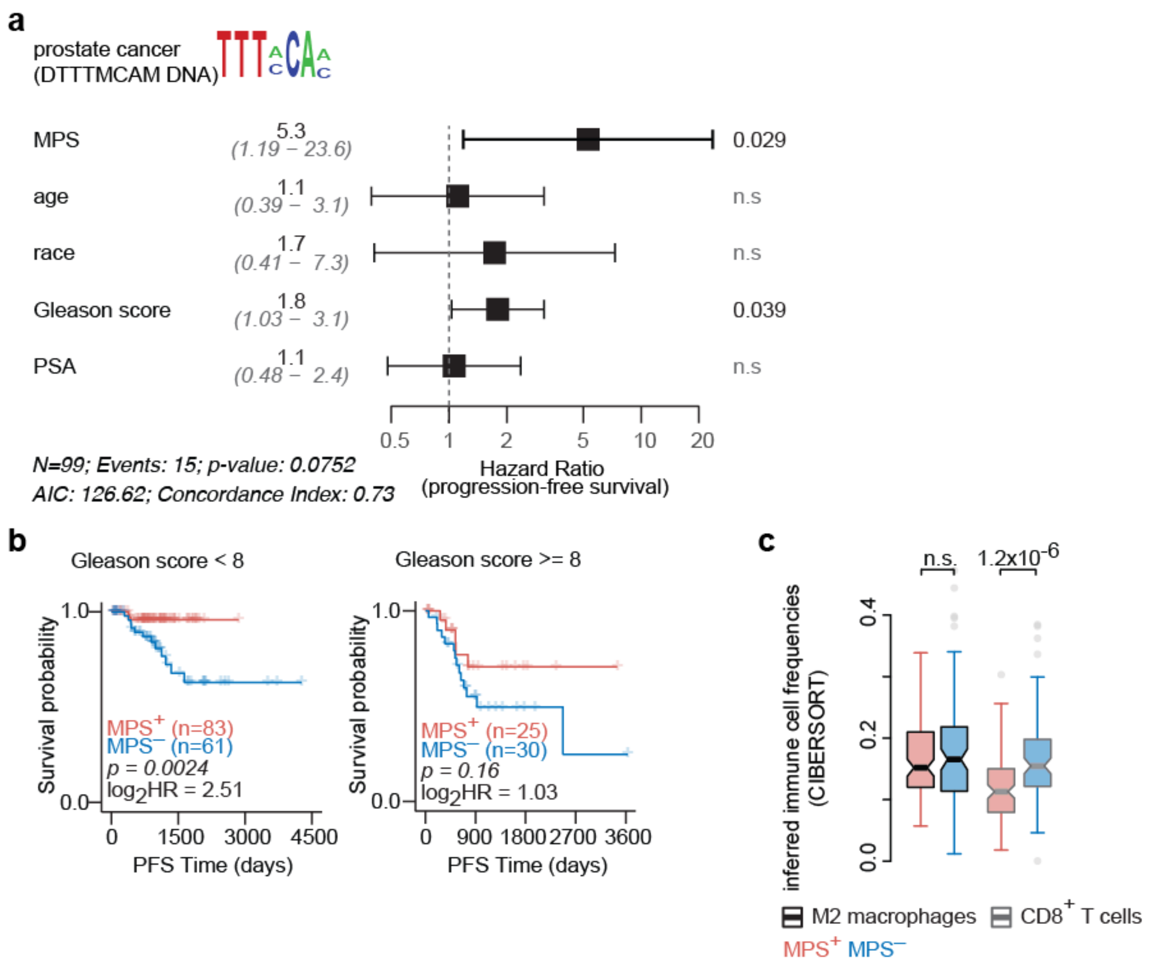
a
prostate cancer
(DTTTMCAM DNA)
MPS
5.3
(1.19 – 23.6)
0.029
age
1.1
(0.39 – 3.1)
n.s
race
1.7
(0.41 – 7.3)
n.s
Gleason score
1.8
(1.03 – 3.1)
0.039
PSA
1.1
(0.48 – 2.4)
n.s
0.5
1
2
5
10
20
Hazard Ratio
(progression-free survival)
N=99; Events: 15; p-value: 0.0752
AIC: 126.62; Concordance Index: 0.73
b
Gleason score < 8
Gleason score >= 8
Survival probability
1.0
0.0
MPS+ (n=83)
MPS− (n=61)
p = 0.0024
log2HR = 2.51
0
1500
3000
4500
PFS Time (days)
MPS+ (n=25)
MPS− (n=30)
p = 0.16
log2HR = 1.03
0
900
1800
2700
3600
c
n.s.
1.2x10-6
inferred immune cell frequencies
(CIBERSORT)
0.0
0.2
0.4
M2 macrophages
CD8+ T cells
MPS+ MPS−

Figure S17. A *de novo* discovered DNA-based *cis*-regulatory module specifies significant prognosis in prostate cancer even after controlling for confounds, Related to Figure 3.
(**a**) Forest plot for prostate cancer patients with a module corresponding to transcripts carrying at least one instance of the DNA motif DTTTMCAM (logo shown) within the first 1KB of their promoters. Shown are results from multivariate cox analysis incorporating module perturbation scores (MPS), age, race, Gleason score and prostate specific antigen (PSA) levels. (**b**) MPS-based patient stratification for this PCM (previous panel) in specified sub-cohorts of the prostate cancer patients including patients whose tumors have Gleason score less than 8 (left panel) and Gleason score that is 8 and above (right panel). Module activation (MPS^+; red) was associated with better overall survival outcomes than module repression ($MPS^–$; blue). (**c**) Boxplots show the inferred frequencies of M2 Macrophages and $CD8^+$ T cells (CIBERSORT[13]) in prostate cancer patients with significant activation (MPS^+; red) or repression ($MPS^–$; blue) of the module associated with the DNA motif DTTTMCAM (module in (**a**-**b**)). One-sided Mann-Whitney test *p*-values are indicated.

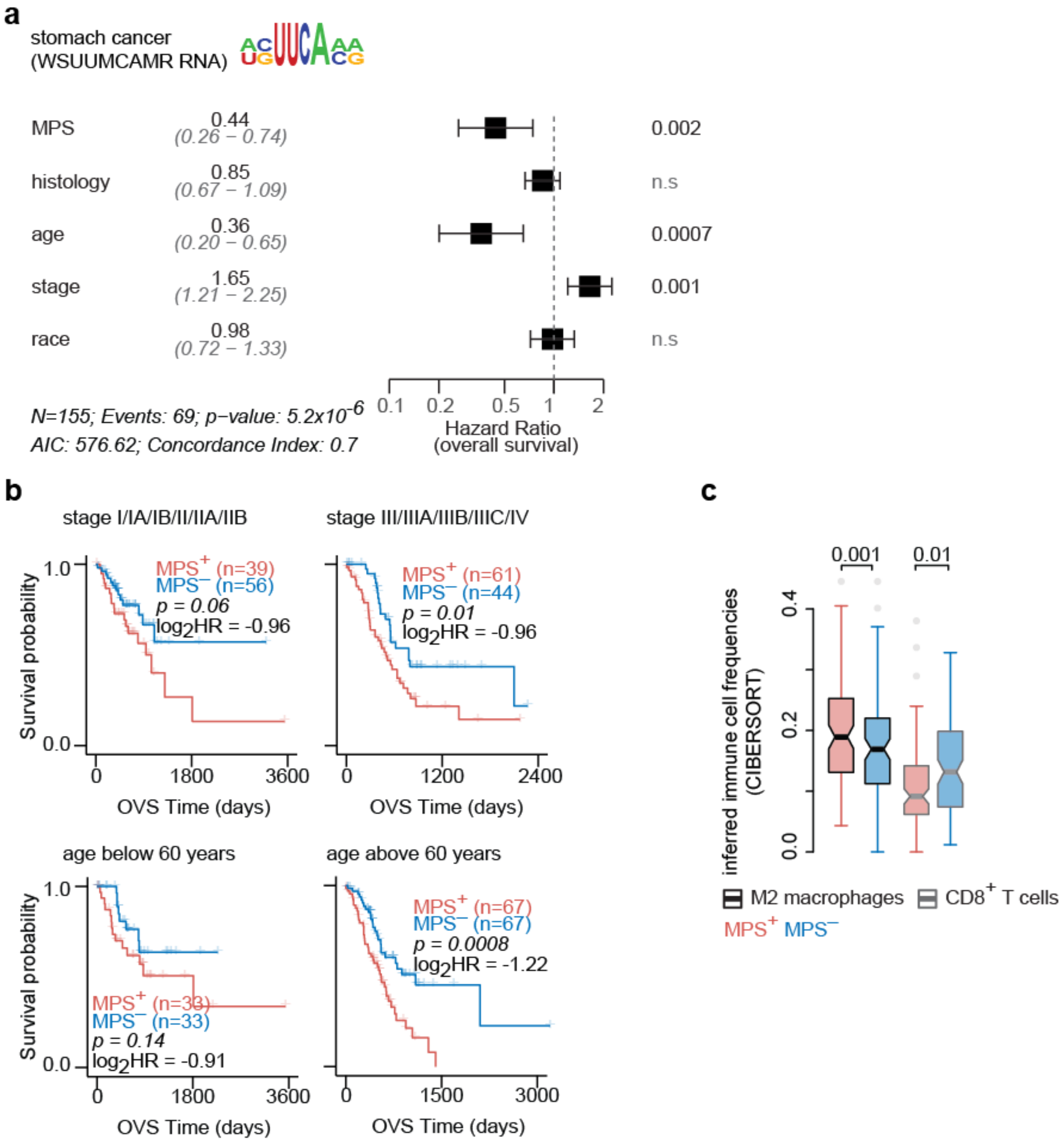
a
stomach cancer
(WSUUMCAMR RNA)
MPS
0.44
(0.26 – 0.74)
0.002
histology
0.85
(0.67 – 1.09)
n.s
age
0.36
(0.20 – 0.65)
0.0007
stage
1.65
(1.21 – 2.25)
0.001
race
0.98
(0.72 – 1.33)
n.s
N=155; Events: 69; p−value: 5.2x10^-6
AIC: 576.62; Concordance Index: 0.7
0.1 0.2 0.5 1 2
Hazard Ratio
(overall survival)
b
stage I/IA/IB/II/IIA/IIB
MPS+ (n=39)
MPS− (n=56)
p = 0.06
log2HR = -0.96
stage III/IIIA/IIIB/IIIC/IV
MPS+ (n=61)
MPS− (n=44)
p = 0.01
log2HR = -0.96
age below 60 years
MPS+ (n=33)
MPS− (n=33)
p = 0.14
log2HR = -0.91
age above 60 years
MPS+ (n=67)
MPS− (n=67)
p = 0.0008
log2HR = -1.22
Survival probability
OVS Time (days)
c
0.001
0.01
inferred immune cell frequencies
(CIBERSORT)
M2 macrophages
CD8+ T cells
MPS+ MPS−

Figure S18. A *de novo* discovered linear RNA-based *cis*-regulatory module specifies significant prognosis in stomach cancer even after controlling for confounds, Related to Figure 3.

(**a**) Forest plot for stomach cancer patients with a module corresponding to transcripts carrying at least one instance of the linear RNA motif WSUUCAMR (logo shown) within the first 1KB of their 3'UTRs. Shown are results from multivariate cox analysis incorporating module perturbation scores (MPS), histological types, age, stage and race. (**b**) MPS-based patient stratification for this PCM (previous panel) in specified sub-cohorts of the stomach cancer patients including patients whose tumors are stage I or stage II (top; left panel), stage III or stage IV (top; right panel), patients whose age at diagnosis is below 60 years (bottom; left panel) or above 60 years (bottom; right panel). Module activation (MPS^+; red) was associated with worse overall survival outcomes than module repression ($MPS^–$; blue). (**c**) Boxplots show the inferred frequencies of M2 Macrophages and $CD8^+$ T cells (CIBERSORT[13]) in stomach cancer patients with significant activation (MPS^+; red) or repression ($MPS^–$; blue) of the module associated with the RNA motif WSUUCAMR (module in (**a**-**b**)). One-sided Mann-Whitney test *p*-values are indicated.

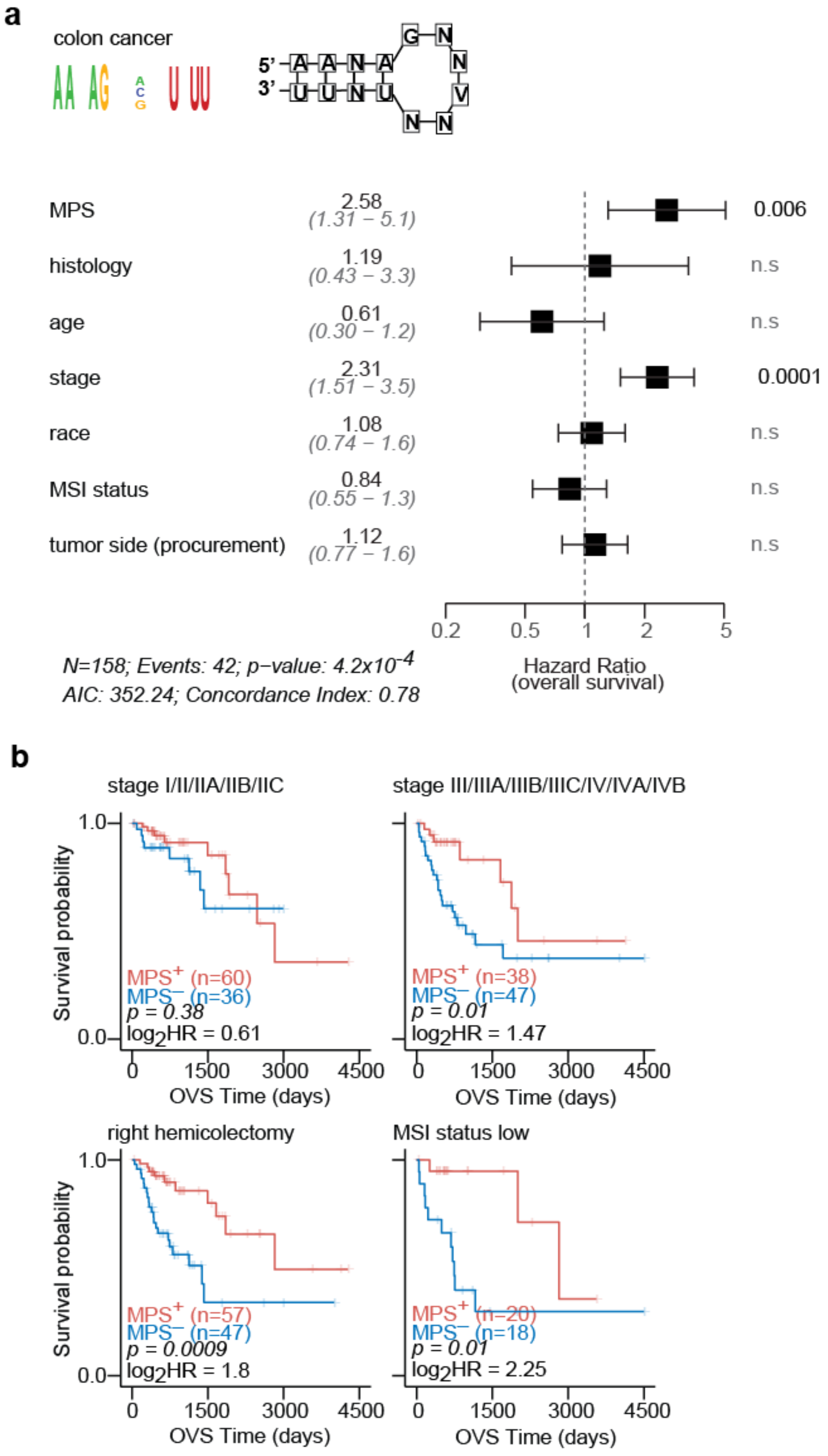
a
colon cancer
5'
3'
MPS
histology
age
stage
race
MSI status
tumor side (procurement)
2.58 (1.31 – 5.1)
1.19 (0.43 – 3.3)
0.61 (0.30 – 1.2)
2.31 (1.51 – 3.5)
1.08 (0.74 – 1.6)
0.84 (0.55 – 1.3)
1.12 (0.77 – 1.6)
0.006
n.s
n.s
0.0001
n.s
n.s
n.s
0.2 0.5 1 2 5
Hazard Ratio (overall survival)
N=158; Events: 42; p−value: 4.2x10^-4
AIC: 352.24; Concordance Index: 0.78
b
stage I/II/IIA/IIB/IIC
MPS+ (n=60)
MPS− (n=36)
p = 0.38
log2HR = 0.61
stage III/IIIA/IIIB/IIIC/IV/IVA/IVB
MPS+ (n=38)
MPS− (n=47)
p = 0.01
log2HR = 1.47
right hemicolectomy
MPS+ (n=57)
MPS− (n=47)
p = 0.0009
log2HR = 1.8
MSI status low
MPS+ (n=20)
MPS− (n=18)
p = 0.01
log2HR = 2.25
Survival probability
OVS Time (days)
0 1500 3000 4500
1.0
0.0

Figure S19. A *de novo* discovered structural RNA-based *cis*-regulatory module specifies significant prognosis in colon cancer even after controlling for confounds, Related to Figure 3.

(**a**) Forest plot for colon cancer patients with a module corresponding to transcripts carrying at least one instance of the structural RNA motif (logo and putative structure shown) within the first 1KB of their 3'UTRs. Shown are results from multivariate cox analysis incorporating module perturbation scores (MPS), histological types, age, stage, race, micro-satellite instability (MSI) status and side of colectomy. (**b**) MPS-based patient stratification for this PCM (previous panel) in specified sub-cohorts of the stomach cancer patients including stage I or stage II tumors (top; left panel), stage III or stage IV tumors (top; right panel), patients who had a right hemicolectomy (bottom; left panel) and tumors characterized to be MSI low (bottom; right panel). Module activation (MPS^+; red) was associated with better overall survival outcomes than module repression (MPS^-; blue). In forest plots, hazard ratios (horizontal axis) with 95% confidence intervals and *p*-values are shown for each variable and in KM plots, statistics of survival comparisons are indicated.

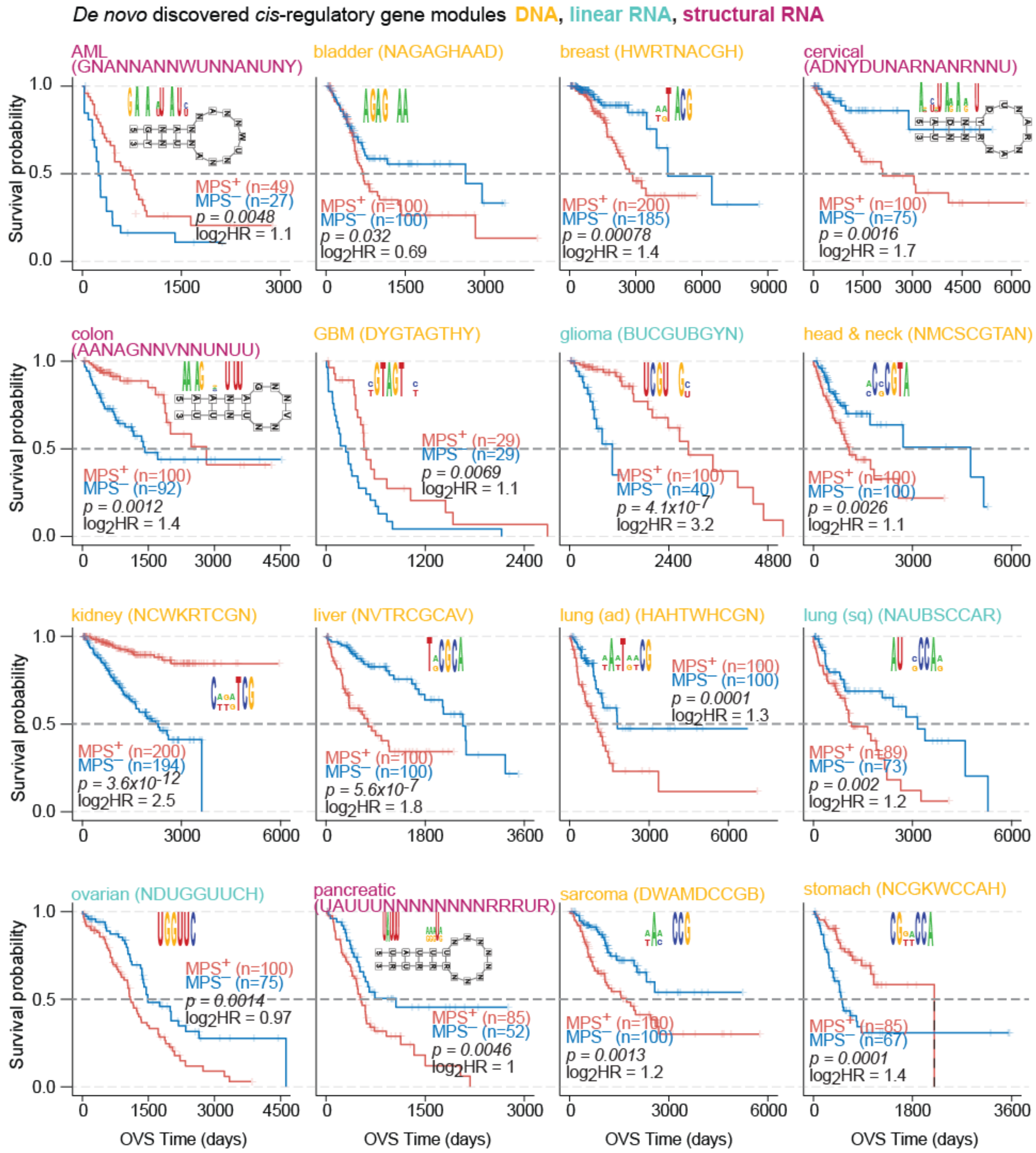
De novo discovered cis-regulatory gene modules DNA, linear RNA, structural RNA
AML (GNANNANNWUNNANUNY)
bladder (NAGAGHAAD)
breast (HWRTNACGH)
cervical (ADNYDUNARNANRNNU)
colon (AANAGNNVNNUNUU)
GBM (DYGTAGTHY)
glioma (BUCGUBGYN)
head & neck (NMCSCGTAN)
kidney (NCWKRTCGN)
liver (NVTRCGCAV)
lung (ad) (HAHTWHCGN)
lung (sq) (NAUBSCCAR)
ovarian (NDUGGUUCH)
pancreatic (UAUUUNNNNNNNNRRRUR)
sarcoma (DWAMDCCGB)
stomach (NCGKWCCAH)
Survival probability
OVS Time (days)
MPS+ (n=49) MPS− (n=27) p = 0.0048 log2HR = 1.1
MPS+ (n=100) MPS− (n=100) p = 0.032 log2HR = 0.69
MPS+ (n=200) MPS− (n=185) p = 0.00078 log2HR = 1.4
MPS+ (n=100) MPS− (n=75) p = 0.0016 log2HR = 1.7
MPS+ (n=100) MPS− (n=92) p = 0.0012 log2HR = 1.4
MPS+ (n=29) MPS− (n=29) p = 0.0069 log2HR = 1.1
MPS+ (n=100) MPS− (n=40) p = 4.1x10-7 log2HR = 3.2
MPS+ (n=100) MPS− (n=100) p = 0.0026 log2HR = 1.1
MPS+ (n=200) MPS− (n=194) p = 3.6x10-12 log2HR = 2.5
MPS+ (n=100) MPS− (n=100) p = 5.6x10-7 log2HR = 1.8
MPS+ (n=100) MPS− (n=100) p = 0.0001 log2HR = 1.3
MPS+ (n=89) MPS− (n=73) p = 0.002 log2HR = 1.2
MPS+ (n=100) MPS− (n=75) p = 0.0014 log2HR = 0.97
MPS+ (n=85) MPS− (n=52) p = 0.0046 log2HR = 1
MPS+ (n=100) MPS− (n=100) p = 0.0013 log2HR = 1.2
MPS+ (n=85) MPS− (n=67) p = 0.0001 log2HR = 1.4

Figure S20. Subset of highly significant *de novo* discovered PCMs for overall survival, Related to STAR Methods, Data Sheet 4.

Kaplan-Meier (KM) plots showcase highly significant *de novo* discovered *cis*-regulatory PCMs for overall survival (OVS) in individual cohorts (labels shown on top). The colors of labels correspond to the categories of the modules- DNA (yellow), linear RNA (green) and structural RNA (purple). KM plots show fraction of surviving patients (Y-axis) and time in months (X-axis) as well as statistics of the comparisons between patients in whom the module is significantly activated (MPS^+) and repressed (MPS^-). The modules were selected such that the associated motifs have no similarities (analyses using TOMOTOM[14]) to previously known transcription factor or RNA binding protein binding sequences or micro-RNA seeds (Methods).

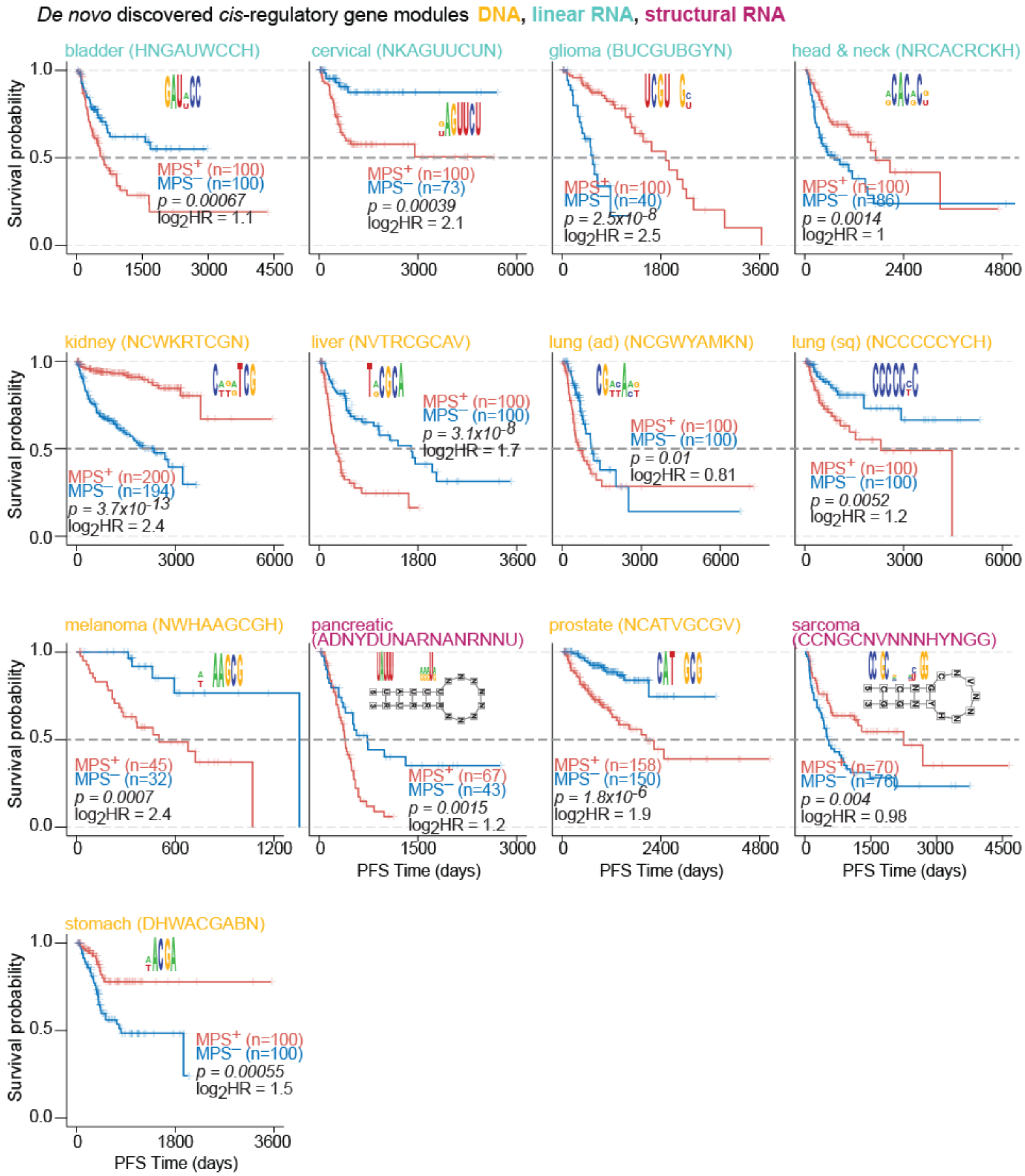
De novo discovered cis-regulatory gene modules DNA, linear RNA, structural RNA
bladder (HNGAUWCCH)
cervical (NKAGUUCUN)
glioma (BUCGUBGYN)
head & neck (NRCACRCKH)
kidney (NCWKRTCGN)
liver (NVTRCGCAV)
lung (ad) (NCGWYAMKN)
lung (sq) (NCCCCCYCH)
melanoma (NWHAAGCGH)
pancreatic (ADNYDUNARNANRNNU)
prostate (NCATVGCGV)
sarcoma (CCNGCNVNNNHYNGG)
stomach (DHWACGABN)
Survival probability
PFS Time (days)
MPS+ (n=100) MPS− (n=100) p = 0.00067 log2HR = 1.1
MPS+ (n=100) MPS− (n=73) p = 0.00039 log2HR = 2.1
MPS+ (n=100) MPS− (n=40) p = 2.5x10-8 log2HR = 2.5
MPS+ (n=100) MPS− (n=86) p = 0.0014 log2HR = 1
MPS+ (n=200) MPS− (n=194) p = 3.7x10-13 log2HR = 2.4
MPS+ (n=100) MPS− (n=100) p = 3.1x10-8 log2HR = 1.7
MPS+ (n=100) MPS− (n=100) p = 0.01 log2HR = 0.81
MPS+ (n=100) MPS− (n=100) p = 0.0052 log2HR = 1.2
MPS+ (n=45) MPS− (n=32) p = 0.0007 log2HR = 2.4
MPS+ (n=67) MPS− (n=43) p = 0.0015 log2HR = 1.2
MPS+ (n=158) MPS− (n=150) p = 1.8x10-6 log2HR = 1.9
MPS+ (n=70) MPS− (n=76) p = 0.004 log2HR = 0.98
MPS+ (n=100) MPS− (n=100) p = 0.00055 log2HR = 1.5

Figure S21. Subset of highly significant *de novo* discovered PCMs for progression-free interval survival, Related to STAR Methods, Data Sheet 4.

Kaplan-Meier (KM) plots showcase highly significant *de novo* discovered *cis*-regulatory PCMs for progression-free interval survival (PFS) in individual cohorts (labels shown on top). The colors of labels correspond to the categories of the modules- DNA (yellow), linear RNA (green) and structural RNA (purple). KM plots show fraction of surviving patients (Y-axis) and time in months (X-axis) as well as statistics of the comparisons between patients in whom the module is significantly activated (MPS^{+}) and repressed (MPS^{-}). The modules were selected such that the associated motifs have no similarities (analyses using TOMOTOM[14]) to previously known transcription factor or RNA binding protein binding sequences or micro-RNA seeds (Methods).

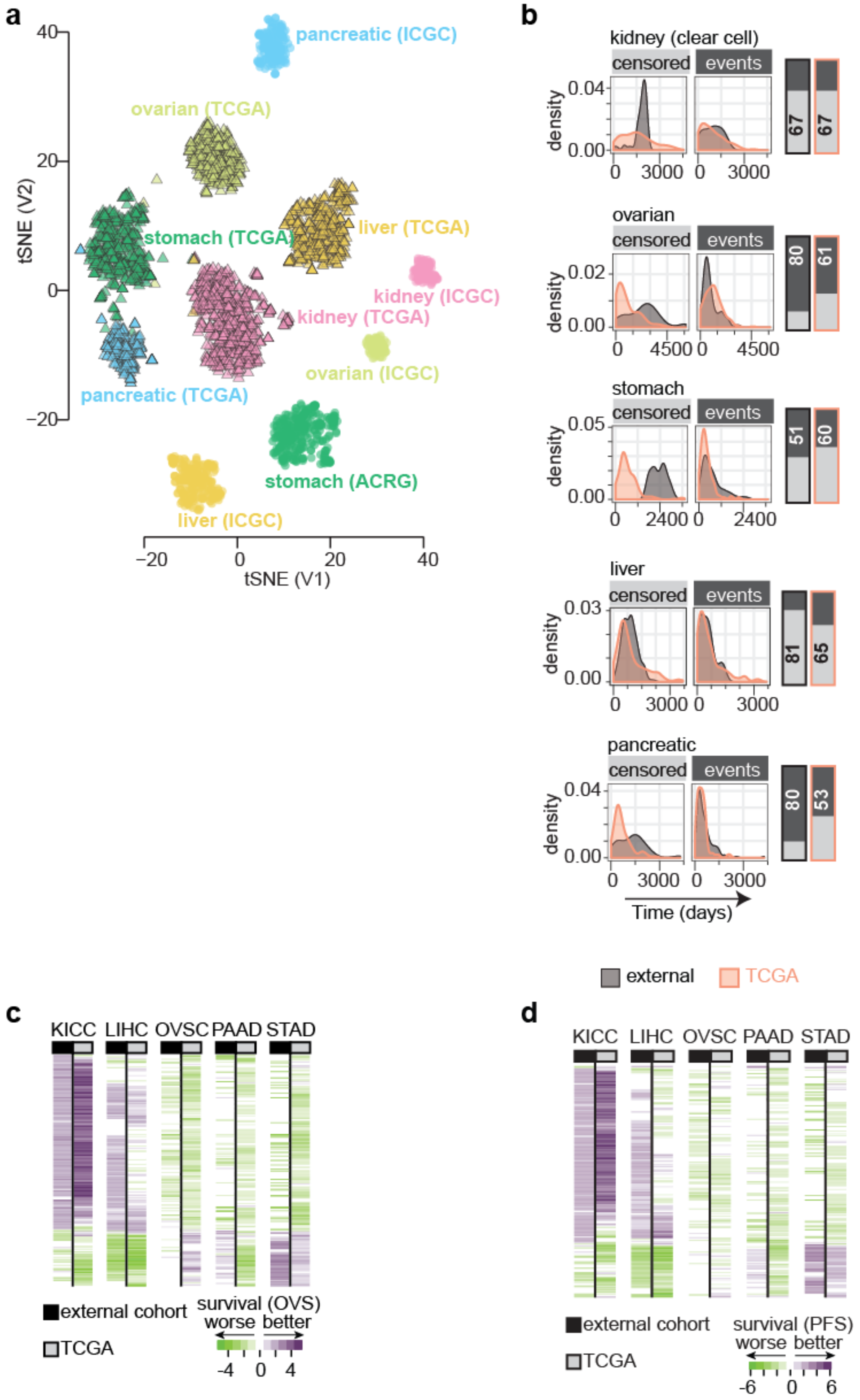
a
pancreatic (ICGC)
ovarian (TCGA)
stomach (TCGA)
liver (TCGA)
kidney (ICGC)
kidney (TCGA)
ovarian (ICGC)
pancreatic (TCGA)
stomach (ACRG)
liver (ICGC)
tSNE (V1)
tSNE (V2)
b
kidney (clear cell)
ovarian
stomach
liver
pancreatic
censored
events
density
Time (days)
external
TCGA
c
KICC LIHC OVSC PAAD STAD
external cohort
TCGA
survival (OVS)
worse better
d
KICC LIHC OVSC PAAD STAD
external cohort
TCGA
survival (PFS)
worse better

Figure S22. Conserved prognostic potential of module perturbations in external cohorts, Related to STAR Methods.

(**a**) Visualization of high-dimensional data using tSNE applied to transcriptome data (z-scored) of primary tumor samples from clear cell kidney cancer (TCGA; n=528 and ICGC-Europe; n=91), liver cancer (TCGA; n=360 and ICGC-Riken; n=223), ovarian cancer (TCGA; n=283 and ICGC-Australia; n=76), pancreatic cancer (TCGA; n=177 and ICGC-Canada; n=163) and stomach cancer (TCGA; n=383 and ACRG; n=300) cohorts. Each dot corresponds to a primary tumor sample from TCGA (triangles) or the external cohort (dots) from tissue-matched cohorts (color, labels shown). (**b**) Clinical characteristics of tissue-matched primary tumors analyzed through TCGA or independent external cohorts. Density plots show the distribution of follow-up times for patients that are censored (light gray; label on top) or event times for the remainder of the cohort (dark gray; label on top). Stacked barplots show percent of events (dark gray) and patients that are censored (light gray) in the external cohort (black) or in TCGA (orange) for liver, ovarian, stomach, clear cell kidney and pancreatic cancer cohorts. (**c**-**d**) Standardized significance of MPS based patient survival in external cohorts (from ICGC and ACRG) and TCGA cohorts (labels indicated on top) of clear cell kidney cancer (KICC), liver cancer (LIHC), ovarian cancer (OVSC), pancreatic cancer (PAAD) and stomach cancer (STAD) samples. Modules that specify recurrent prognostic value in the external cohorts are shown and the values (color key indicated) correspond to the Wald statistic computed using the Cox proportional-hazards model for (**c**) overall survival and (**d**) progression-free interval survival.

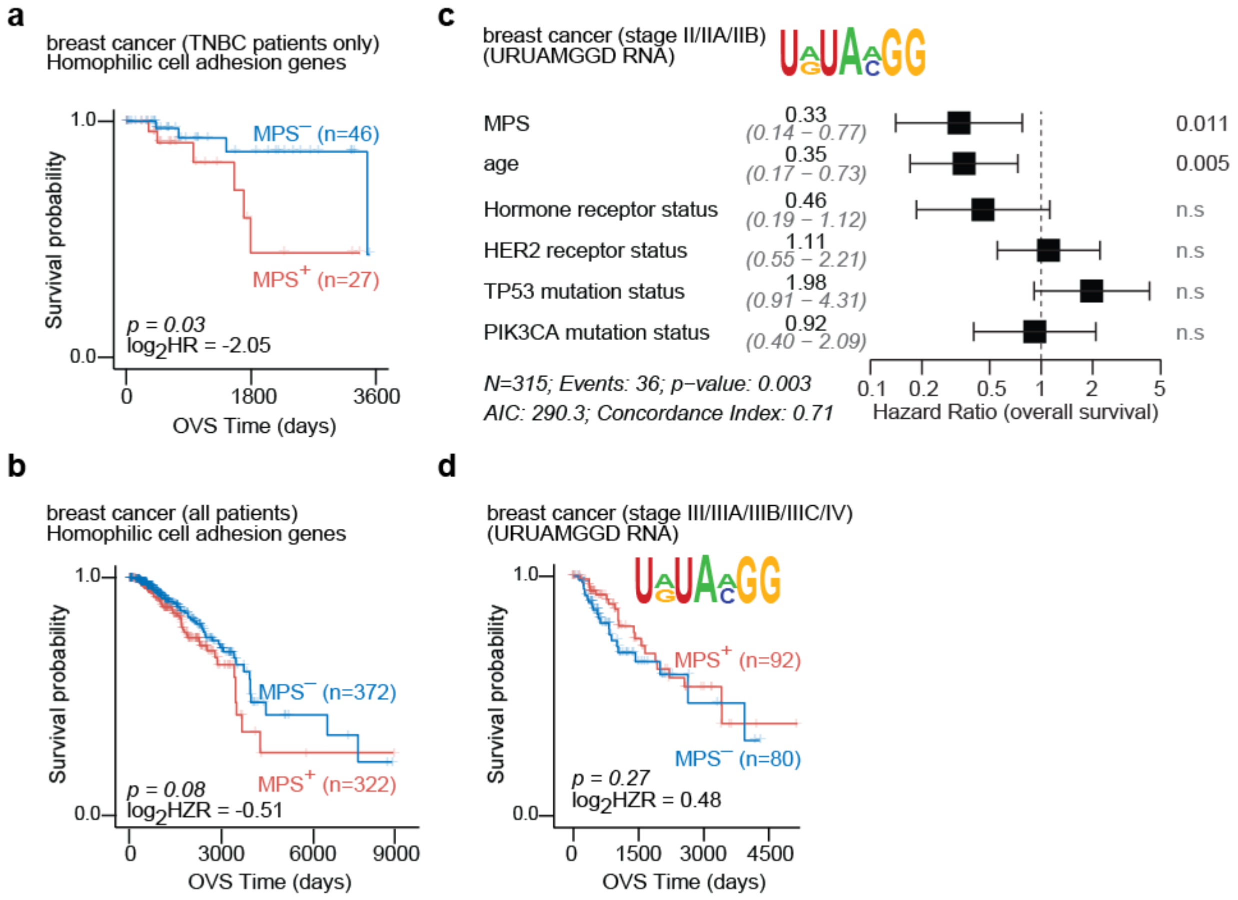
a
breast cancer (TNBC patients only)
Homophilic cell adhesion genes
MPS− (n=46)
MPS+ (n=27)
p = 0.03
log2HR = -2.05
Survival probability
1.0
0.0
0
1800
3600
OVS Time (days)
c
breast cancer (stage II/IIA/IIB)
(URUAMGGD RNA)
MPS 0.33 (0.14 − 0.77) 0.011
age 0.35 (0.17 − 0.73) 0.005
Hormone receptor status 0.46 (0.19 − 1.12) n.s
HER2 receptor status 1.11 (0.55 − 2.21) n.s
TP53 mutation status 1.98 (0.91 − 4.31) n.s
PIK3CA mutation status 0.92 (0.40 − 2.09) n.s
N=315; Events: 36; p−value: 0.003
AIC: 290.3; Concordance Index: 0.71
0.1 0.2 0.5 1 2 5
Hazard Ratio (overall survival)
b
breast cancer (all patients)
Homophilic cell adhesion genes
MPS− (n=372)
MPS+ (n=322)
p = 0.08
log2HZR = -0.51
Survival probability
1.0
0.0
0
3000
6000
9000
OVS Time (days)
d
breast cancer (stage III/IIIA/IIIB/IIIC/IV)
(URUAMGGD RNA)
MPS+ (n=92)
MPS− (n=80)
p = 0.27
log2HZR = 0.48
Survival probability
1.0
0.0
0
1500
3000
4500
OVS Time (days)

Figure S23. ***A priori*** **incorporation of histological types and tumor stage identifies significant prognostic modules within the specified sub-cohorts, Related to Figure 4, Data Sheet 6.**

(**a**) In triple negative breast cancer patients, significant activation of module (MPS^+; red) corresponding to homophilic cell adhesion specified worse prognosis than patients with significant module repression ($MPS^–$; blue). (**b**) On the full breast cancer cohort, the module corresponding to homophilic cell adhesion (MSigDB[2] Gene Ontology; 173 genes) did not stratify patients based on overall survival. (**c**) Forest plot for stage II/IIA/IIB breast cancer patients with a *de novo* discovered *cis*-regulatory gene module corresponding to transcripts carrying at least one instance of the linear RNA motif URUAMGGD (logo shown; 1082 genes) within the first 1KB of their 3'UTRs. Shown are results from multivariate cox analysis incorporating module perturbation scores (MPS), patient age, tumor stage, PIK3CA and TP53 mutation status, hormone receptor status and HER2 status. For overall survival prognosis, hazard ratios (horizontal axis) with 95% confidence intervals and *p*-values are shown for each predictor. (**d**) For this module (previous panel), stratification of breast cancer patients with stage III/IIIA/IIIB/IIIC/IV cancers into MPS^+ and $MPS^–$ groups did not provide significantly different survival trajectories.

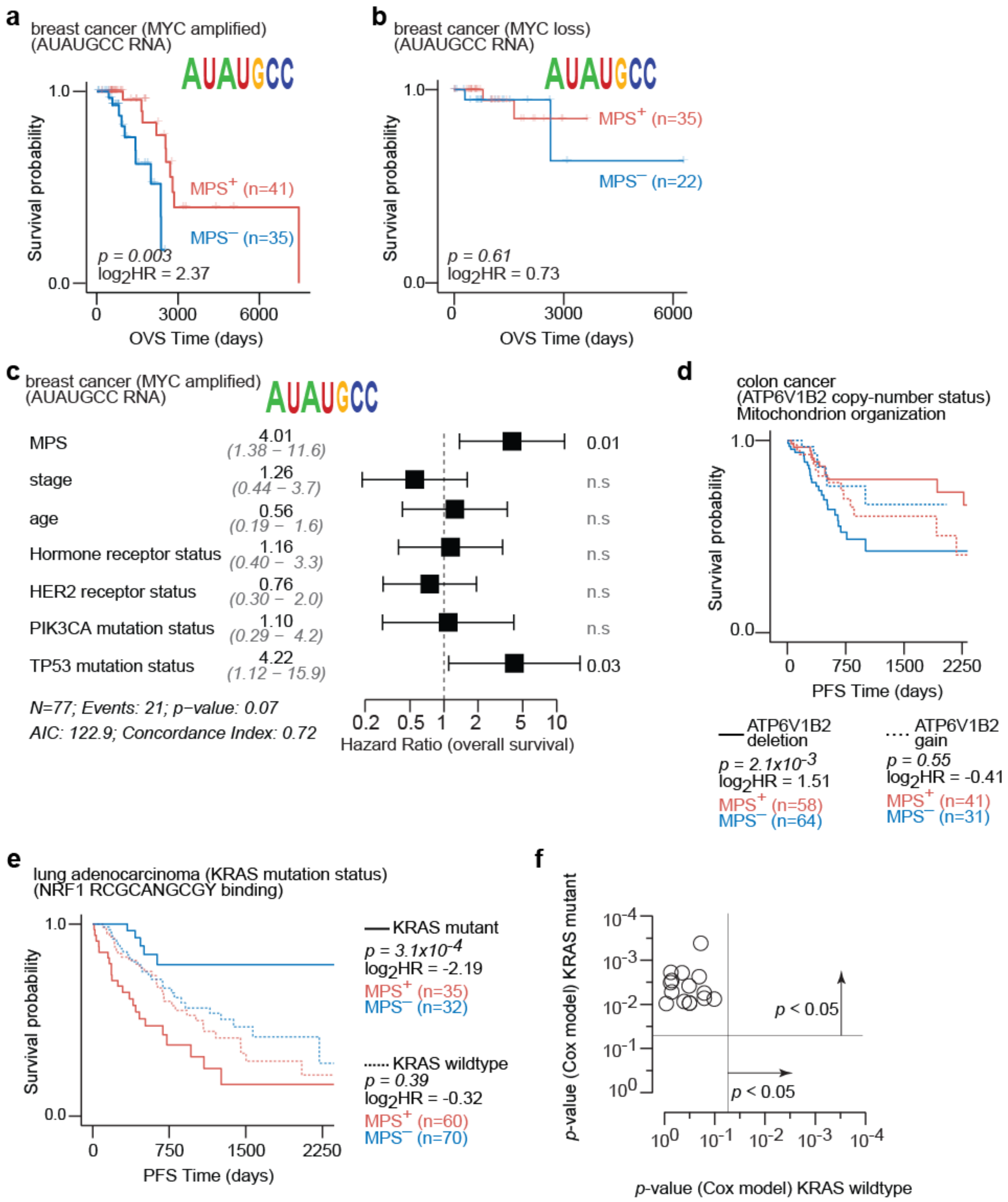
a
breast cancer (MYC amplified)
(AUAUGCC RNA)
AUAUGCC
Survival probability
MPS+ (n=41)
MPS− (n=35)
p = 0.003
log2HR = 2.37
OVS Time (days)
b
breast cancer (MYC loss)
(AUAUGCC RNA)
AUAUGCC
Survival probability
MPS+ (n=35)
MPS− (n=22)
p = 0.61
log2HR = 0.73
OVS Time (days)
c
breast cancer (MYC amplified)
(AUAUGCC RNA)
AUAUGCC
MPS 4.01 (1.38 − 11.6) 0.01
stage 1.26 (0.44 − 3.7) n.s
age 0.56 (0.19 − 1.6) n.s
Hormone receptor status 1.16 (0.40 − 3.3) n.s
HER2 receptor status 0.76 (0.30 − 2.0) n.s
PIK3CA mutation status 1.10 (0.29 − 4.2) n.s
TP53 mutation status 4.22 (1.12 − 15.9) 0.03
N=77; Events: 21; p−value: 0.07
AIC: 122.9; Concordance Index: 0.72
Hazard Ratio (overall survival)
d
colon cancer
(ATP6V1B2 copy-number status)
Mitochondrion organization
Survival probability
PFS Time (days)
ATP6V1B2 deletion
p = 2.1x10-3
log2HR = 1.51
MPS+ (n=58)
MPS− (n=64)
ATP6V1B2 gain
p = 0.55
log2HR = -0.41
MPS+ (n=41)
MPS− (n=31)
e
lung adenocarcinoma (KRAS mutation status)
(NRF1 RCGCANGCGY binding)
Survival probability
PFS Time (days)
KRAS mutant
p = 3.1x10-4
log2HR = -2.19
MPS+ (n=35)
MPS− (n=32)
KRAS wildtype
p = 0.39
log2HR = -0.32
MPS+ (n=60)
MPS− (n=70)
f
p-value (Cox model) KRAS mutant
p < 0.05
p < 0.05
p-value (Cox model) KRAS wildtype

Figure S24. *A priori* incorporation of prominent genomic aberrations identifies significant prognostic modules within the specified sub-cohorts, Related to Figure 4, Data Sheet 6. (**a**) Breast cancer patients with deep amplification of MYC (Methods) and with significant module activation (MPS$^+$; red) for transcripts harboring at least one instance of the RNA motif AUAUGCC (logo shown; 529 genes) within the first 1KB of their 3'UTRs, showed better overall survival (OVS) prognosis than samples with significant module-repression (MPS$^–$; blue). (**b**) For this module (previous panel), stratification of breast cancer patient tumors that harbor deletions or deep deletions at the MYC locus (Methods) into MPS$^+$ and MPS$^–$ groups did not provide significantly different survival trajectories. (**c**) Forest plot for breast cancer patients harboring deep amplification of the locus spanning MYC (Methods) with a *de novo* discovered *cis*-regulatory gene module corresponding to transcripts carrying at least one instance of the linear RNA motif AUAUGCC (logo shown) within the first 1KB of their 3'UTRs. Shown are results from multivariate cox analysis incorporating module perturbation scores (MPS), patient age, tumor stage, PIK3CA and TP53 mutation status, hormone receptor status and HER2 status. For overall survival prognosis, hazard ratios (horizontal axis) with 95% confidence intervals and *p*-values are shown for each predictor. (**d**) Colon cancer tumor samples harboring deletions at the ATP6V1B2 locus with significant activation of the module corresponding to genes annotated to be involved in mitochondrion organization (MSigDB[2]; 509 genes) showed better progression-free interval survival (PFS) that samples with significant repression. For this module, stratification of colon cancer patients harboring amplification or deep amplification of the ATP6V1B2 locus into MPS$^+$ and MPS$^–$ groups did not provide significantly different survival trajectories (dashed lines). (**e**) Lung adenocarcinoma patients with mutated KRAS and with significant module activation (MPS$^+$; red) for genes harboring at least one instance of binding sites for NRF1 transcription factor (MSigDB[2] M9394; 958 genes) showed worse prognosis than patients with significant module repression (MPS$^–$; blue). For this module, stratification of lung adenocarcinoma patients that are wildtype for KRAS into MPS$^+$ and MPS$^–$ groups did not provide significantly different survival trajectories (dashed lines). (**f**) Survival stratifications in lung adenocarcinoma patients harboring mutated or wildtype KRAS based on perturbation scores of modules identified to be significantly prognostic in patients whose tumors harbor mutated KRAS. Scatterplot shows comparisons between *p*-values (Cox proportional hazards model) in survival stratification (MPS$^+$ vs. MPS$^–$) of lung adenocarcinoma patients harboring wildtype (X-axis) and mutated (Y-axis) KRAS.

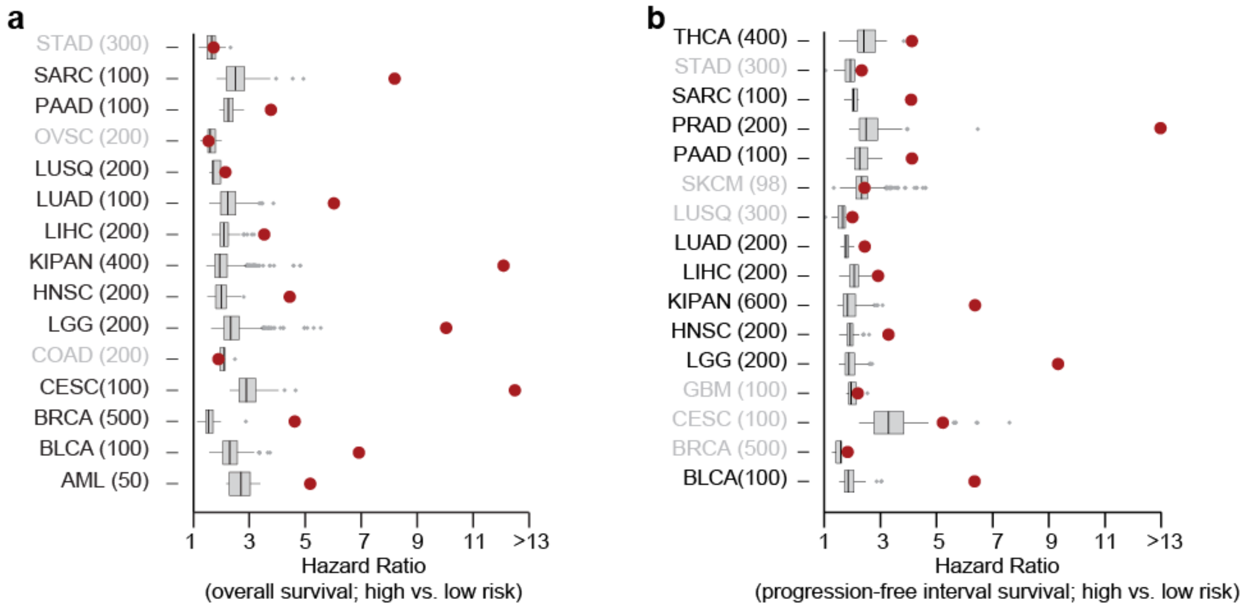

a
STAD (300)
SARC (100)
PAAD (100)
OVSC (200)
LUSQ (200)
LUAD (100)
LIHC (200)
KIPAN (400)
HNSC (200)
LGG (200)
COAD (200)
CESC(100)
BRCA (500)
BLCA (100)
AML (50)
1 3 5 7 9 11 >13
Hazard Ratio
(overall survival; high vs. low risk)
b
THCA (400)
STAD (300)
SARC (100)
PRAD (200)
PAAD (100)
SKCM (98)
LUSQ (300)
LUAD (200)
LIHC (200)
KIPAN (600)
HNSC (200)
LGG (200)
GBM (100)
CESC (100)
BRCA (500)
BLCA(100)
1 3 5 7 9 11 >13
Hazard Ratio
(progression-free interval survival; high vs. low risk)

Figure S25. Predictions from ensemble learning models that utilize combinations of PCMs provide stronger patient stratification than individual PCMs, Related to Figure 5, STAR Methods.

(**a-b**) Hazard ratio distributions obtained by stratifying patients using the perturbation scores of prognostic cancer modules in individual cohorts. Red dots correspond to patient stratifications derived from random survival forest models[15] trained on PCMs for (**a**) overall survival (OVS) or (**b**) progression-free interval survival (PFS) in individual cohorts (see Methods). Cohorts in which the random survival forest models provided stronger stratification compared to every individual PCM are in bold font and number of samples in each survival comparison is indicated.

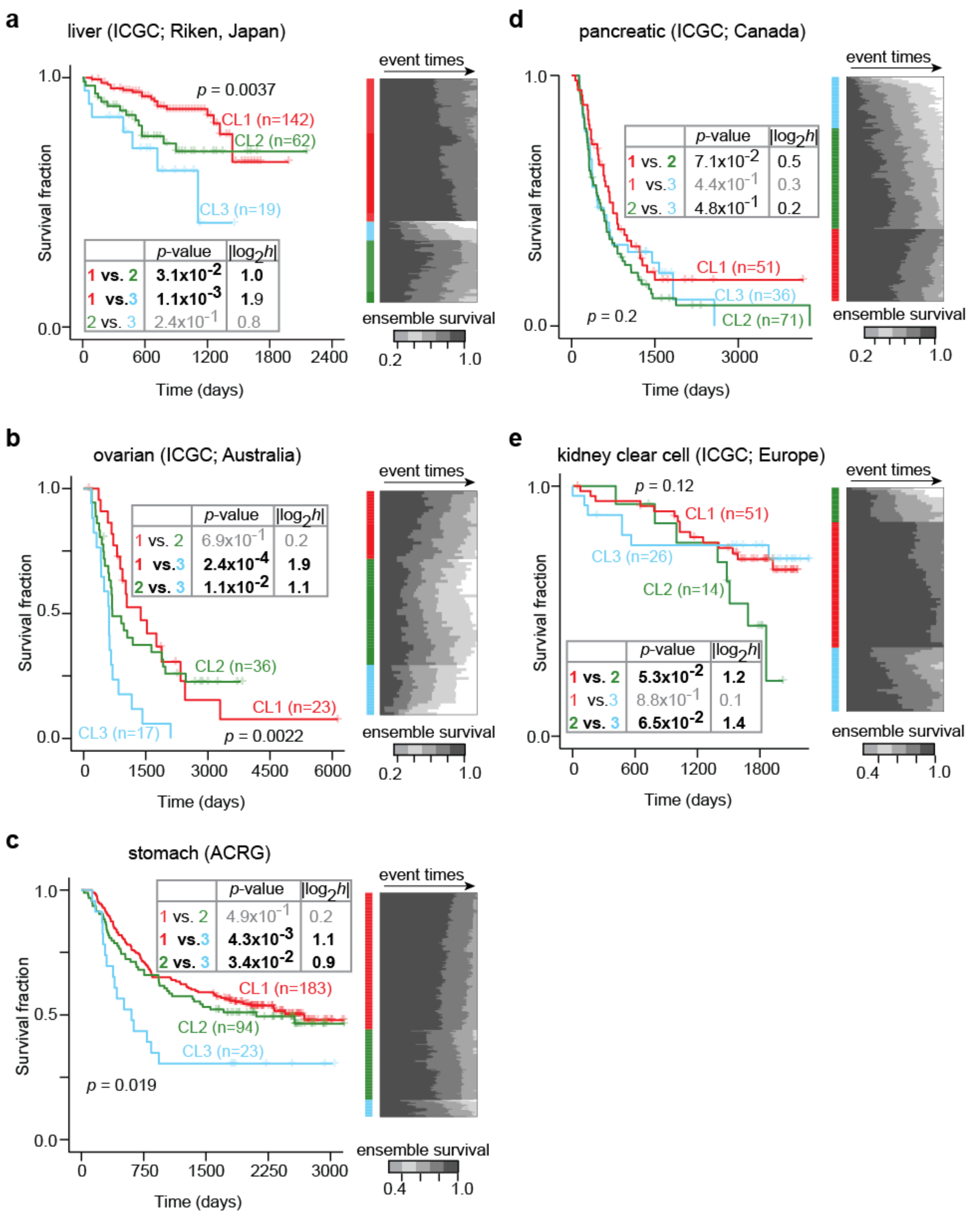
a
liver (ICGC; Riken, Japan)
p = 0.0037
CL1 (n=142)
CL2 (n=62)
CL3 (n=19)
p-value |log2h|
1 vs. 2 3.1x10-2 1.0
1 vs. 3 1.1x10-3 1.9
2 vs. 3 2.4x10-1 0.8
Survival fraction
Time (days)
event times
ensemble survival
d
pancreatic (ICGC; Canada)
p-value |log2h|
1 vs. 2 7.1x10-2 0.5
1 vs. 3 4.4x10-1 0.3
2 vs. 3 4.8x10-1 0.2
CL1 (n=51)
CL3 (n=36)
CL2 (n=71)
p = 0.2
b
ovarian (ICGC; Australia)
p-value |log2h|
1 vs. 2 6.9x10-1 0.2
1 vs. 3 2.4x10-4 1.9
2 vs. 3 1.1x10-2 1.1
CL2 (n=36)
CL1 (n=23)
CL3 (n=17)
p = 0.0022
e
kidney clear cell (ICGC; Europe)
p = 0.12
CL1 (n=51)
CL3 (n=26)
CL2 (n=14)
p-value |log2h|
1 vs. 2 5.3x10-2 1.2
1 vs. 3 8.8x10-1 0.1
2 vs. 3 6.5x10-2 1.4
c
stomach (ACRG)
p-value |log2h|
1 vs. 2 4.9x10-1 0.2
1 vs. 3 4.3x10-3 1.1
2 vs. 3 3.4x10-2 0.9
CL1 (n=183)
CL2 (n=94)
CL3 (n=23)
p = 0.019

Figure S26. Predictions from ensemble learning models that utilize combinations of PCMs are conserved in tissue-matched external cohorts, Related to STAR Methods.

(**a-e**) Random survival forest models trained on TCGA data are used to make survival predictions on the full set of patients in tissue-matched external cohorts. The predicted ensemble survival probabilities of individual patients at event times (unique death times) are shown (heatmap; right panel). Rows (patients) are clustered using k-means clustering with Euclidean distance and the optimal cluster size (3; NbClust[16]) is chosen based on Krzanowski and Lai[17], Calinski and Harbasz[18], silhouette[19] and gap statistic[20] metrics. Kaplan-Meier plots (left panel) show the survival trajectories of patients in these clusters (colors indicated). Across the cohorts, there was clear concordance between predicted survival probabilities and survival trajectories of patients in the cohorts. Statistics for survival comparison as well as pairwise survival comparisons are listed for (**a**) liver, (**b**) ovarian, (**c**) stomach, (**d**) pancreatic and (**e**) clear cell kidney cancer cohorts.

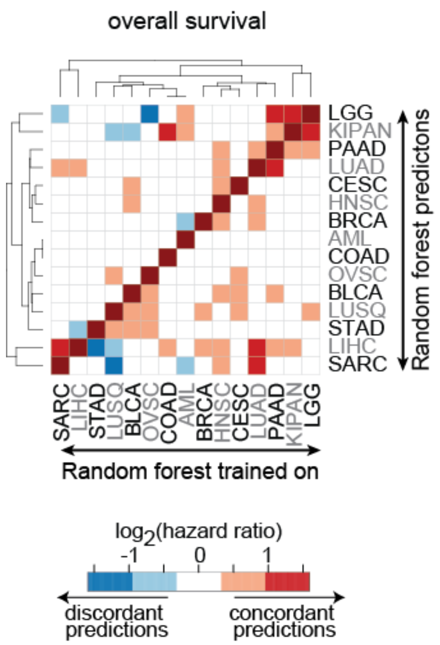
overall survival
LGG
KIPAN
PAAD
LUAD
CESC
HNSC
BRCA
AML
COAD
OVSC
BLCA
LUSQ
STAD
LIHC
SARC
Random forest predictons
SARC
LIHC
STAD
LUSQ
BLCA
OVSC
COAD
AML
BRCA
HNSC
CESC
LUAD
PAAD
KIPAN
LGG
Random forest trained on
log2(hazard ratio)
-1
0
1
discordant predictions
concordant predictions

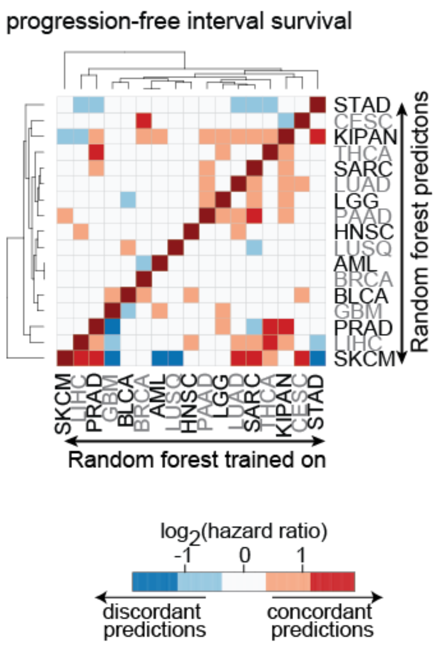
progression-free interval survival
STAD
CESC
KIPAN
THCA
SARC
LUAD
LGG
PAAD
HNSC
LUSQ
AML
BRCA
BLCA
GBM
PRAD
LIHC
SKCM
Random forest predictons
SKCM
LIHC
PRAD
GBM
BLCA
BRCA
AML
LUSQ
HNSC
PAAD
LGG
LUAD
SARC
THCA
KIPAN
CESC
STAD
Random forest trained on
log2(hazard ratio)
-1
0
1
discordant predictions
concordant predictions

Figure S27. Predictions from ensemble learning models that utilize combinations of PCMs are conserved in some cancers despite diverse tissues-of-origin, Related to STAR Methods.

For all possible pairs of cohorts, the prognostic predictive value of random survival forest models trained on PCMs in one cohort (columns) was tested on every other cohort (rows). Survival comparisons in the test cohort were made based on predicted risk (top and bottom 40%). Colors correspond to hazard ratios and indicate if predictions in the test cohorts exhibit concordance (red) or discordance (blue) with training cohort. Diagonal entries of the heatmap (same training and test cohort and thus, concordant predictions) are in dark red.

a

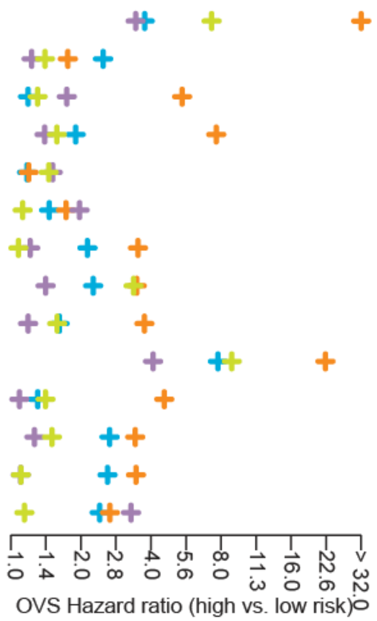

standard clinical factors | SNVs | CNAs | MPS

	MPS vs. standard clinical factors	MPS vs. SNVs	MPS vs. CNAs
THYM (50)	$9.6x10^{-3}$	$9.6x10^{-3}$	$2.4x10^{-4}$
STAD (100)	n.s	$1.4x10^{-2}$	$1.0x10^{-4}$
SARC (100)	$5.4x10^{-6}$	$5.4x10^{-6}$	$5.4x10^{-6}$
PAAD (50)	$5.4x10^{-6}$	$5.4x10^{-6}$	$5.4x10^{-6}$
OVSC (100)	n.s	n.s	n.s
LUSQ (100)	$1.2x10^{-2}$	$3.8x10^{-5}$	n.s
LUAD (100)	$3.8x10^{-5}$	$5.4x10^{-6}$	$5.4x10^{-6}$
LIHC (100)	$2.2x10^{-5}$	n.s	$5.4x10^{-6}$
HNSC (100)	$5.4x10^{-6}$	$5.4x10^{-6}$	$5.4x10^{-6}$
LGG (100)	$5.4x10^{-6}$	$1.6x10^{-4}$	$5.4x10^{-6}$
CESC (100)	$5.4x10^{-6}$	$5.4x10^{-6}$	$5.4x10^{-6}$
BRCA (500)	$3.4x10^{-3}$	$5.4x10^{-6}$	$5.4x10^{-6}$
BLCA (50)	$7.3x10^{-3}$	$5.4x10^{-6}$	$5.4x10^{-6}$
AML (50)	n.s	$5.4x10^{-6}$	n.s

b

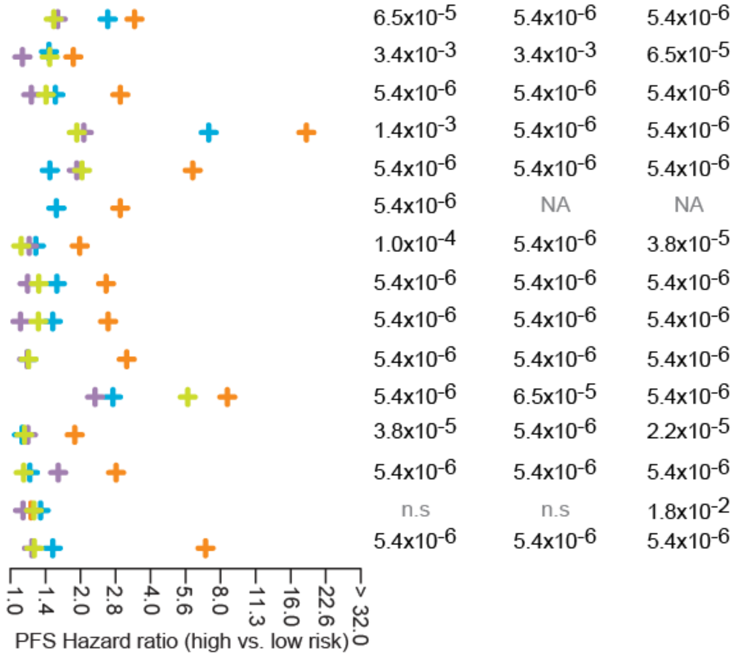

standard clinical factors | SNVs | CNAs | MPS

	MPS vs. standard clinical factors	MPS vs. SNVs	MPS vs. CNAs
THCA (200)	$6.5x10^{-5}$	$5.4x10^{-6}$	$5.4x10^{-6}$
STAD (100)	$3.4x10^{-3}$	$3.4x10^{-3}$	$6.5x10^{-5}$
SARC (100)	$5.4x10^{-6}$	$5.4x10^{-6}$	$5.4x10^{-6}$
PRAD(100)	$1.4x10^{-3}$	$5.4x10^{-6}$	$5.4x10^{-6}$
PAAD (50)	$5.4x10^{-6}$	$5.4x10^{-6}$	$5.4x10^{-6}$
SKCM (50)	$5.4x10^{-6}$	NA	NA
LUSQ (100)	$1.0x10^{-4}$	$5.4x10^{-6}$	$3.8x10^{-5}$
LUAD (100)	$5.4x10^{-6}$	$5.4x10^{-6}$	$5.4x10^{-6}$
LIHC (100)	$5.4x10^{-6}$	$5.4x10^{-6}$	$5.4x10^{-6}$
HNSC (100)	$5.4x10^{-6}$	$5.4x10^{-6}$	$5.4x10^{-6}$
LGG (100)	$5.4x10^{-6}$	$6.5x10^{-5}$	$5.4x10^{-6}$
GBM (50)	$3.8x10^{-5}$	$5.4x10^{-6}$	$2.2x10^{-5}$
CESC (100)	$5.4x10^{-6}$	$5.4x10^{-6}$	$5.4x10^{-6}$
BRCA (500)	n.s	n.s	$1.8x10^{-2}$
BLCA (50)	$5.4x10^{-6}$	$5.4x10^{-6}$	$5.4x10^{-6}$

Figure S28. Random survival forest models trained on module perturbations provide more prognostic value than models trained on conventional clinical factors, Related to Figure 5, STAR Methods.

Performance of random survival forest models trained on standard clinical factors (blue), module perturbation scores (orange), recurrent single-nucleotide variants (SNV; green) and prominent copy-number aberrations (CNA; purple). For each cohort (rows), random forest models were trained in 10-fold cross-validation using prognostic cancer modules (PCMs) in each cohort for (**a**) overall survival (OVS) and (**b**) progression-free interval survival (PFS). Hazard ratios were obtained using Cox proportional hazards model on patients stratified into high and low risk groups based on predictions from the random survival forest models[15]. Number of patients used in each comparison is specified. Crosses correspond to the median hazard ratio calculated from 10 repetitions of the random survival forest predictions with randomized initializations. One-sided Mann-Whitney test *p*-values for comparisons of hazard ratios from models trained on MPS and each of standard clinical factors, SNVs and CNAs are also indicated for each cohort.

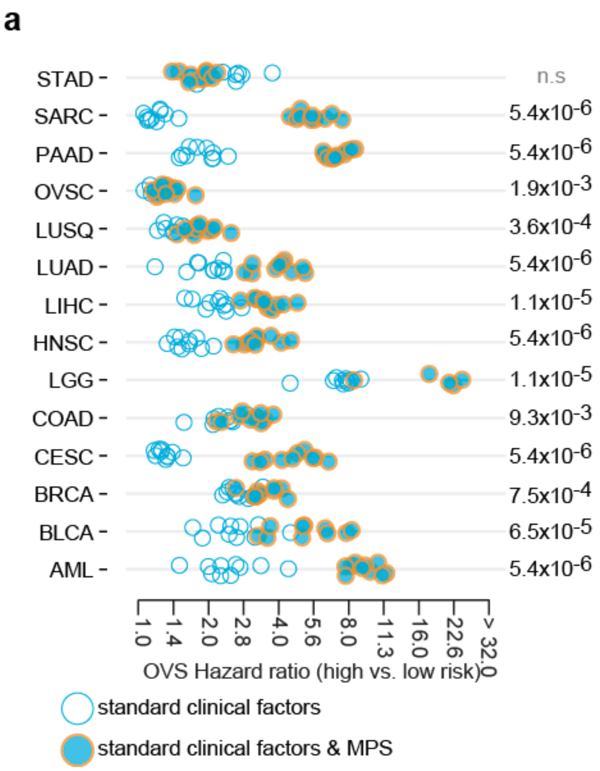
a
STAD
SARC
PAAD
OVSC
LUSQ
LUAD
LIHC
HNSC
LGG
COAD
CESC
BRCA
BLCA
AML
n.s
5.4x10-6
5.4x10-6
1.9x10-3
3.6x10-4
5.4x10-6
1.1x10-5
5.4x10-6
1.1x10-5
9.3x10-3
5.4x10-6
7.5x10-4
6.5x10-5
5.4x10-6
1.0 1.4 2.0 2.8 4.0 5.6 8.0 11.3 16.0 22.6 > 32.0
OVS Hazard ratio (high vs. low risk)
standard clinical factors
standard clinical factors & MPS
b
THCA
STAD
SARC
PRAD
PAAD
SKCM
LUSQ
LUAD
LIHC
HNSC
LGG
GBM
CESC
BRCA
BLCA
5.4x10-6
9.3x10-3
1.1x10-5
5.5x10-5
5.4x10-6
5.4x10-6
6.5x10-5
5.4x10-6
5.4x10-6
5.4x10-6
5.4x10-6
2.2x10-5
5.4x10-6
5.4x10-6
5.4x10-6
1.0 1.4 2.0 2.8 4.0 5.6 8.0 11.3 16.0 22.6 > 32.0
PFS Hazard ratio (high vs. low risk)
standard clinical factors
standard clinical factors & MPS

Figure S29. Random survival forest models trained on module perturbations provide additional prognostic value to models trained on conventional clinical factors, Related to STAR Methods.

(**a**-**b**) Performance of random survival forest models trained on only standard clinical factors (open blue circles) or standard clinical factors and perturbation scores of PCMs (blue dots with orange outline) for (**a**) overall survival (OVS) and, (**b**) progression-free interval survival (PFS). Each dot corresponds to the hazard ratio of a 10-fold cross-validated random survival forest model. For every cohort, distributions of hazard ratios obtained from 10 randomly initialized instances of the random survival forests are shown. Comparisons between hazard ratios obtained from the random survival forest models are shown (*p*-values from one-sided Mann-Whitney test).

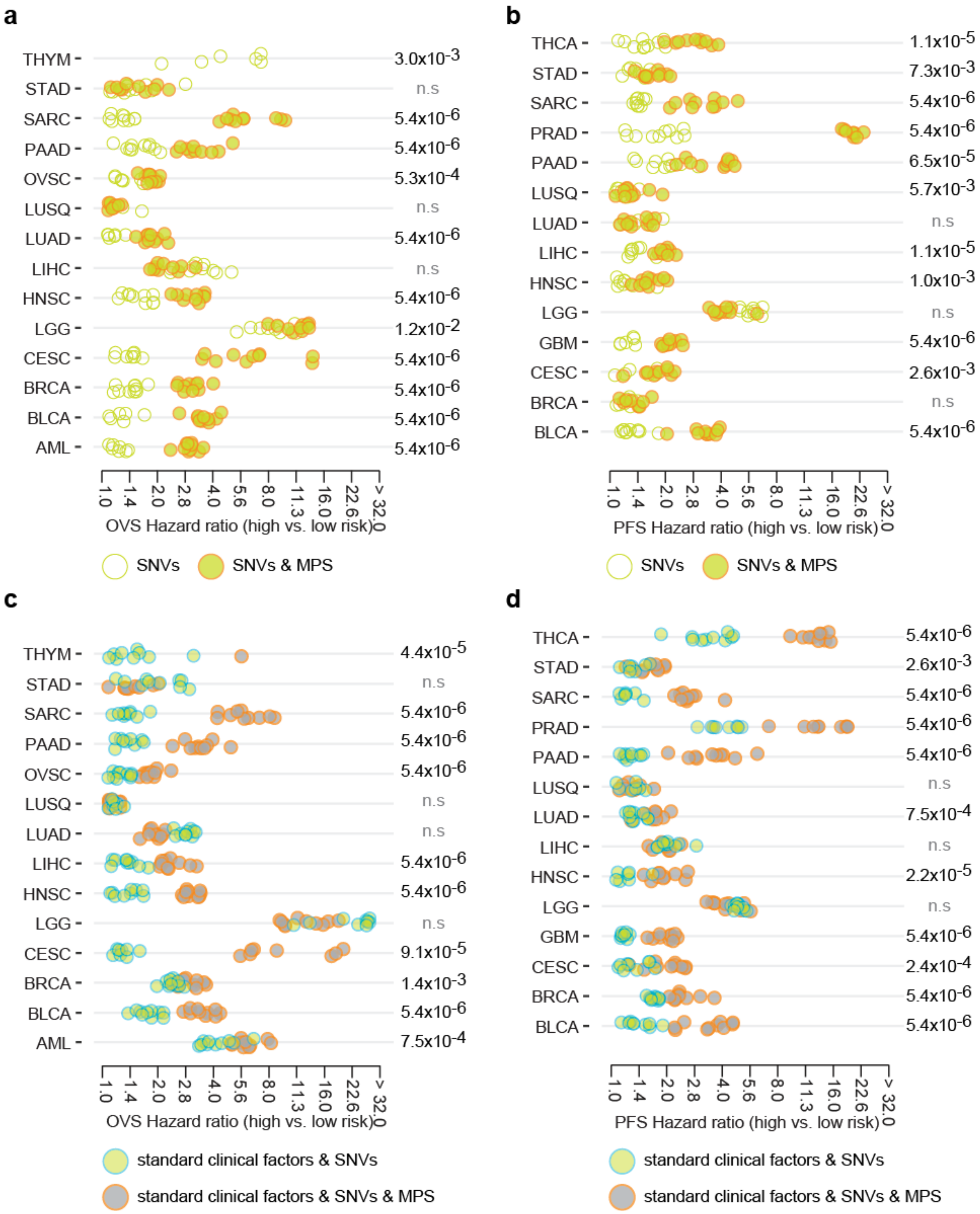
a
THYM
STAD
SARC
PAAD
OVSC
LUSQ
LUAD
LIHC
HNSC
LGG
CESC
BRCA
BLCA
AML
3.0x10-3
n.s
5.4x10-6
5.4x10-6
5.3x10-4
n.s
5.4x10-6
n.s
5.4x10-6
1.2x10-2
5.4x10-6
5.4x10-6
5.4x10-6
5.4x10-6
1.0 1.4 2.0 2.8 4.0 5.6 8.0 11.3 16.0 22.6 > 32.0
OVS Hazard ratio (high vs. low risk)
SNVs
SNVs & MPS
b
THCA
STAD
SARC
PRAD
PAAD
LUSQ
LUAD
LIHC
HNSC
LGG
GBM
CESC
BRCA
BLCA
1.1x10-5
7.3x10-3
5.4x10-6
5.4x10-6
6.5x10-5
5.7x10-3
n.s
1.1x10-5
1.0x10-3
n.s
5.4x10-6
2.6x10-3
n.s
5.4x10-6
1.0 1.4 2.0 2.8 4.0 5.6 8.0 11.3 16.0 22.6 > 32.0
PFS Hazard ratio (high vs. low risk)
SNVs
SNVs & MPS
c
THYM
STAD
SARC
PAAD
OVSC
LUSQ
LUAD
LIHC
HNSC
LGG
CESC
BRCA
BLCA
AML
4.4x10-5
n.s
5.4x10-6
5.4x10-6
5.4x10-6
n.s
n.s
5.4x10-6
5.4x10-6
n.s
9.1x10-5
1.4x10-3
5.4x10-6
7.5x10-4
1.0 1.4 2.0 2.8 4.0 5.6 8.0 11.3 16.0 22.6 > 32.0
OVS Hazard ratio (high vs. low risk)
standard clinical factors & SNVs
standard clinical factors & SNVs & MPS
d
THCA
STAD
SARC
PRAD
PAAD
LUSQ
LUAD
LIHC
HNSC
LGG
GBM
CESC
BRCA
BLCA
5.4x10-6
2.6x10-3
5.4x10-6
5.4x10-6
5.4x10-6
n.s
7.5x10-4
n.s
2.2x10-5
n.s
5.4x10-6
2.4x10-4
5.4x10-6
5.4x10-6
1.0 1.4 2.0 2.8 4.0 5.6 8.0 11.3 16.0 22.6 > 32.0
PFS Hazard ratio (high vs. low risk)
standard clinical factors & SNVs
standard clinical factors & SNVs & MPS

Figure S30. Random survival forests trained on prognostic modules provide significantly improved patient stratification compared to prominent SNVs and standard clinical factors, Related to Figure 5, STAR Methods.

(**a**-**b**) Performance of random survival forest models trained on prominent single-nucleotide variants (SNVs) (open green circles) or SNVs and module perturbation scores of PCMs in each cohort (green dots with orange outline) for (**a**) overall survival (OVS) and, (**b**) progression-free interval survival (PFS). (**c**-**d** Performance of random survival forest models are shown for models trained on prominent SNVs and standard clinical factors with (gray dots with orange outline) or without (green dots with blue outline) module perturbation scores for (**c**) overall survival (OVS) and, (**d**) progression-free interval survival (PFS). Hazard ratios were obtained using Cox proportional hazards model on patients stratified into high and low risk groups based on predictions from the random survival forest models[15]. Each dot corresponds to the predicted hazard ratio of a 10-fold cross-validated random forest model. For every cohort, distributions of hazard ratios obtained from 10 randomly initialized instances are shown. Comparisons between hazard ratios obtained from the two sets of random survival forest models are shown (*p*-values from one-sided Mann-Whitney test).

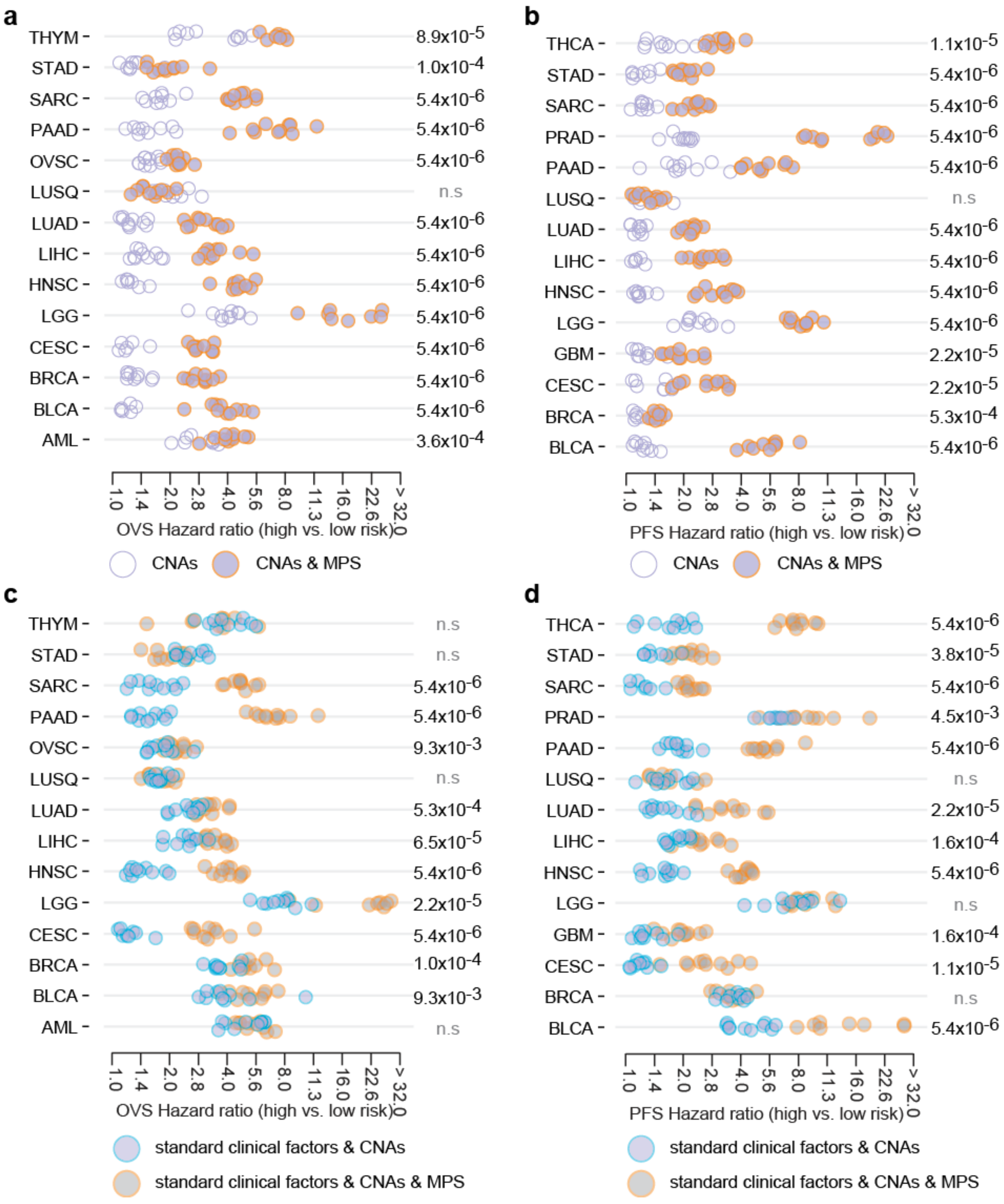
a
THYM
STAD
SARC
PAAD
OVSC
LUSQ
LUAD
LIHC
HNSC
LGG
CESC
BRCA
BLCA
AML
8.9x10-5
1.0x10-4
5.4x10-6
n.s
3.6x10-4
1.0
1.4
2.0
2.8
4.0
5.6
8.0
11.3
16.0
22.6
> 32.0
OVS Hazard ratio (high vs. low risk)
CNAs
CNAs & MPS
b
THCA
STAD
SARC
PRAD
PAAD
LUSQ
LUAD
LIHC
HNSC
LGG
GBM
CESC
BRCA
BLCA
1.1x10-5
5.4x10-6
n.s
2.2x10-5
5.3x10-4
PFS Hazard ratio (high vs. low risk)
CNAs
CNAs & MPS
c
n.s
5.4x10-6
9.3x10-3
5.3x10-4
6.5x10-5
2.2x10-5
1.0x10-4
OVS Hazard ratio (high vs. low risk)
standard clinical factors & CNAs
standard clinical factors & CNAs & MPS
d
5.4x10-6
3.8x10-5
4.5x10-3
n.s
2.2x10-5
1.6x10-4
1.6x10-4
1.1x10-5
PFS Hazard ratio (high vs. low risk)
standard clinical factors & CNAs
standard clinical factors & CNAs & MPS

Figure S31. Random survival forests trained on prognostic modules provide significantly improved patient stratification compared to prominent CNAs and standard clinical factors, Related to STAR Methods.

(**a**-**b**) Performance of random survival forest models trained on prominent copy-number aberrations (CNAs) (open purple circles) or CNAs and module perturbation scores of PCMs in each cohort (purple dots with orange outline) for (**a**) overall survival (OVS) and, (**b**) progression-free interval survival (PFS). (**c**-**d**) Performance of random survival forest models are shown for models trained on prominent CNAs and standard clinical factors) with (gray dots with orange outline) or without (purple dots with blue outline) module perturbation scores for (**c**) overall survival (OVS) and, (**d**) progression-free interval survival (PFS). Hazard ratios were obtained using Cox proportional hazards model on patients stratified into high and low risk groups based on predictions from the random survival forest models[15]. Each dot corresponds to the predicted hazard ratio of a 10-fold cross-validated random forest model. For every cohort, distributions of hazard ratios obtained from 10 randomly initialized instances are shown. Comparisons between hazard ratios obtained from the two sets of random survival forest models are shown (*p*-values from one-sided Mann-Whitney test).

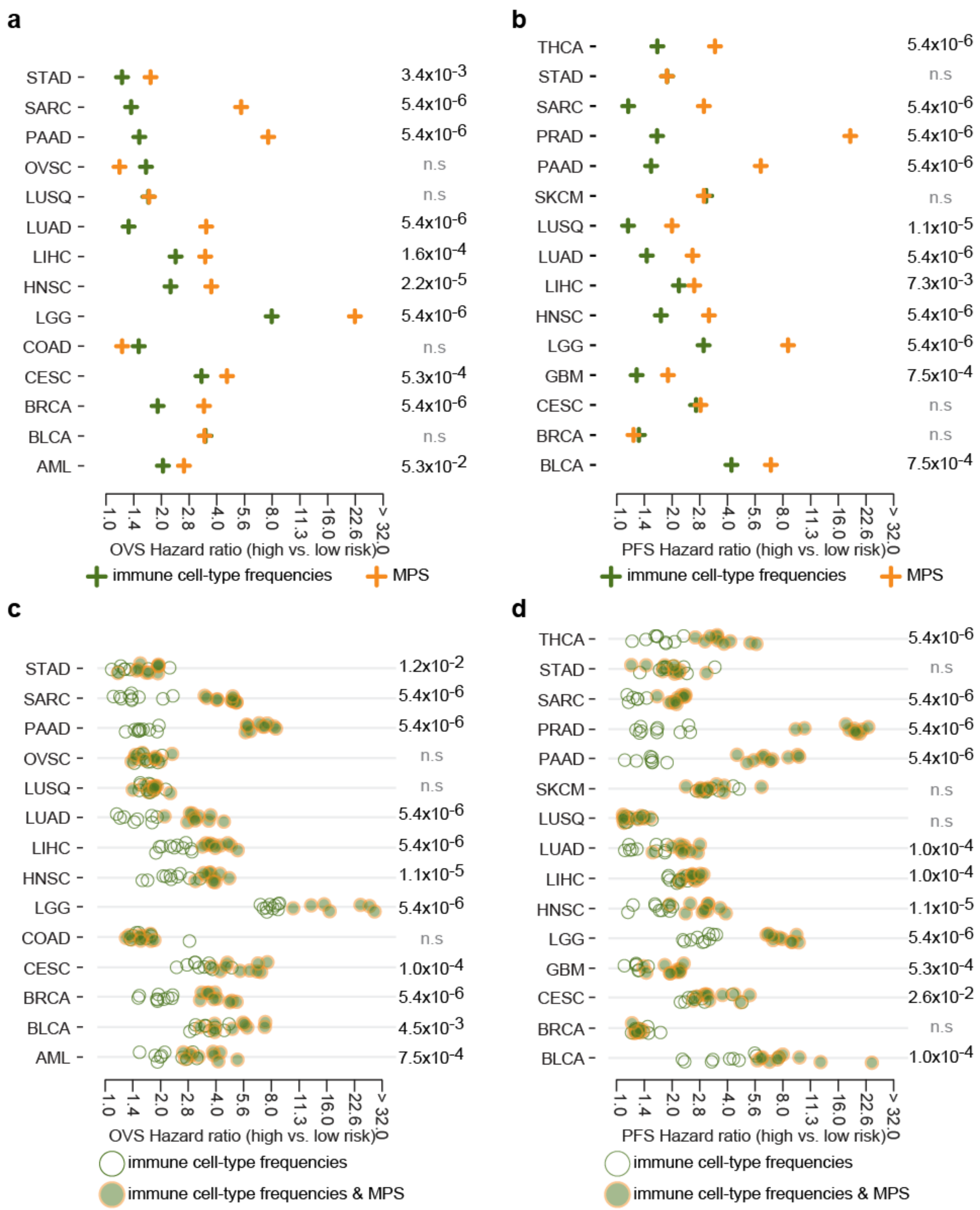
a
STAD
SARC
PAAD
OVSC
LUSQ
LUAD
LIHC
HNSC
LGG
COAD
CESC
BRCA
BLCA
AML
3.4x10-3
5.4x10-6
5.4x10-6
n.s
n.s
5.4x10-6
1.6x10-4
2.2x10-5
5.4x10-6
n.s
5.3x10-4
5.4x10-6
n.s
5.3x10-2
1.0 1.4 2.0 2.8 4.0 5.6 8.0 11.3 16.0 22.6 > 32.0
OVS Hazard ratio (high vs. low risk)
immune cell-type frequencies
MPS
b
THCA
STAD
SARC
PRAD
PAAD
SKCM
LUSQ
LUAD
LIHC
HNSC
LGG
GBM
CESC
BRCA
BLCA
5.4x10-6
n.s
5.4x10-6
5.4x10-6
5.4x10-6
n.s
1.1x10-5
5.4x10-6
7.3x10-3
5.4x10-6
5.4x10-6
7.5x10-4
n.s
n.s
7.5x10-4
1.0 1.4 2.0 2.8 4.0 5.6 8.0 11.3 16.0 22.6 > 32.0
PFS Hazard ratio (high vs. low risk)
immune cell-type frequencies
MPS
c
STAD
SARC
PAAD
OVSC
LUSQ
LUAD
LIHC
HNSC
LGG
COAD
CESC
BRCA
BLCA
AML
1.2x10-2
5.4x10-6
5.4x10-6
n.s
n.s
5.4x10-6
5.4x10-6
1.1x10-5
5.4x10-6
n.s
1.0x10-4
5.4x10-6
4.5x10-3
7.5x10-4
1.0 1.4 2.0 2.8 4.0 5.6 8.0 11.3 16.0 22.6 > 32.0
OVS Hazard ratio (high vs. low risk)
immune cell-type frequencies
immune cell-type frequencies & MPS
d
THCA
STAD
SARC
PRAD
PAAD
SKCM
LUSQ
LUAD
LIHC
HNSC
LGG
GBM
CESC
BRCA
BLCA
5.4x10-6
n.s
5.4x10-6
5.4x10-6
5.4x10-6
n.s
n.s
1.0x10-4
1.0x10-4
1.1x10-5
5.4x10-6
5.3x10-4
2.6x10-2
n.s
1.0x10-4
1.0 1.4 2.0 2.8 4.0 5.6 8.0 11.3 16.0 22.6 > 32.0
PFS Hazard ratio (high vs. low risk)
immune cell-type frequencies
immune cell-type frequencies & MPS

Figure S32. Random survival forests trained on prognostic modules provide significantly improved patient stratification compared to inferred immune cell-type frequencies, Related to STAR Methods.

(**a**-**b**) Performance of random survival forest models trained on module perturbation scores of PCMs (orange) and inferred immune cell-type frequencies (dark green). For each cohort (rows), random survival forest models were trained in a 10-fold cross-validated setup for (**a**) overall survival (OVS) and (**b**) progression-free interval survival (PFS). Crosses correspond to the median hazard ratios calculated from 10 repetitions of the random forest predictions with randomized initializations. (**c**-**d**) Performance of random survival forest models are shown for random survival forest models trained on prominent inferred immune cell-type frequencies (open dark green circles) or inferred immune cell-type frequencies and PCMs (dark green dots with orange outlines) for (**c**) overall survival (OVS) and, (**d**) progression-free interval survival (PFS). Hazard ratios were obtained using Cox proportional hazards model on patients stratified into high and low risk groups[15]. Each dot corresponds to the hazard ratio of a 10-fold cross-validated random forest model. For every cohort, distributions of hazard ratios obtained from 10 randomly initialized instances are shown. Comparisons between hazard ratios obtained from the two sets of random survival forest models are shown (*p*-values from one-sided Mann-Whitney test).

References

1. Chakravarty, D., Gao, J., Phillips, S., Kundra, R., Zhang, H., Wang, J., Rudolph, J.E., Yaeger, R., Soumerai, T., Nissan, M.H., et al. (2017). OncoKB: A Precision Oncology Knowledge Base. JCO Precis. Oncol. *1*, 1–16. 10.1200/PO.17.00011.

2. Liberzon, A., Birger, C., Thorvaldsdóttir, H., Ghandi, M., Mesirov, J.P., and Tamayo, P. (2015). The Molecular Signatures Database Hallmark Gene Set Collection. Cell Syst. *1*, 417–425. 10.1016/j.cels.2015.12.004.

3. Bryne, J.C., Valen, E., Tang, M.-H.E., Marstrand, T., Winther, O., da Piedade, I., Krogh, A., Lenhard, B., and Sandelin, A. (2008). JASPAR, the open access database of transcription factor-binding profiles: new content and tools in the 2008 update. Nucleic Acids Res. *36*, D102-106. 10.1093/nar/gkm955.

4. Ray, D., Kazan, H., Cook, K.B., Weirauch, M.T., Najafabadi, H.S., Li, X., Gueroussov, S., Albu, M., Zheng, H., Yang, A., et al. (2013). A compendium of RNA-binding motifs for decoding gene regulation. Nature *499*, 172–177. 10.1038/nature12311.

5. Weirauch, M.T., Yang, A., Albu, M., Cote, A.G., Montenegro-Montero, A., Drewe, P., Najafabadi, H.S., Lambert, S.A., Mann, I., Cook, K., et al. (2014). Determination and inference of eukaryotic transcription factor sequence specificity. Cell *158*, 1431–1443. 10.1016/j.cell.2014.08.009.

6. Elemento, O., Slonim, N., and Tavazoie, S. (2007). A universal framework for regulatory element discovery across all genomes and data types. Mol. Cell *28*, 337–350. 10.1016/j.molcel.2007.09.027.

7. Goodarzi, H., Najafabadi, H.S., Oikonomou, P., Greco, T.M., Fish, L., Salavati, R., Cristea, I.M., and Tavazoie, S. (2012). Systematic discovery of structural elements governing stability of mammalian messenger RNAs. Nature *485*, 264–268. 10.1038/nature11013.

8. Drier, Y., Sheffer, M., and Domany, E. (2013). Pathway-based personalized analysis of cancer. Proc. Natl. Acad. Sci. *110*, 6388–6393. 10.1073/pnas.1219651110.

9. Martin, M.L., Zeng, Z., Adileh, M., Jacobo, A., Li, C., Vakiani, E., Hua, G., Zhang, L., Haimovitz-Friedman, A., Fuks, Z., et al. (2018). Logarithmic expansion of LGR5+ cells in human colorectal cancer. Cell. Signal. *42*, 97–105. 10.1016/j.cellsig.2017.09.018.

10. Liberzon, A., Birger, C., Thorvaldsdóttir, H., Ghandi, M., Mesirov, J.P., and Tamayo, P. (2015). The Molecular Signatures Database Hallmark Gene Set Collection. Cell Syst. *1*, 417–425. 10.1016/j.cels.2015.12.004.

11. Drier, Y. (2017). pathifier. 10.18129/B9.BIOC.PATHIFIER.

12. Frey, B.J., and Dueck, D. (2007). Clustering by Passing Messages Between Data Points. Science *315*, 972–976. 10.1126/science.1136800.

13. Newman, A.M., Liu, C.L., Green, M.R., Gentles, A.J., Feng, W., Xu, Y., Hoang, C.D., Diehn, M., and Alizadeh, A.A. (2015). Robust enumeration of cell subsets from tissue expression profiles. Nat. Methods *12*, 453–457. 10.1038/nmeth.3337.

14. Gupta, S., Stamatoyannopoulos, J.A., Bailey, T.L., and Noble, W.S. (2007). Quantifying similarity between motifs. Genome Biol. *8*, R24. 10.1186/gb-2007-8-2-r24.

15. Ishwaran, H., Kogalur, U.B., Blackstone, E.H., and Lauer, M.S. (2008). Random survival forests. Ann. Appl. Stat. *2*, 841–860. 10.1214/08-AOAS169.

16. Charrad, M., Ghazzali, N., Boiteau, V., and Niknafs, A. (2014). **NbClust** : An *R* Package for Determining the Relevant Number of Clusters in a Data Set. J. Stat. Softw. *61*. 10.18637/jss.v061.i06.

17. Krzanowski, W.J., and Lai, Y.T. (1988). A Criterion for Determining the Number of Groups in a Data Set Using Sum-of-Squares Clustering. Biometrics *44*, 23. 10.2307/2531893.

18. Calinski, T., and Harabasz, J. (1974). A dendrite method for cluster analysis. Commun. Stat. - Theory Methods *3*, 1–27. 10.1080/03610927408827101.

19. Rousseeuw, P.J. (1987). Silhouettes: A graphical aid to the interpretation and validation of cluster analysis. J. Comput. Appl. Math. *20*, 53–65. 10.1016/0377-0427(87)90125-7.

20. Tibshirani, R., Walther, G., and Hastie, T. (2001). Estimating the number of clusters in a data set via the gap statistic. J. R. Stat. Soc. Ser. B Stat. Methodol. *63*, 411–423. 10.1111/1467-9868.00293.
